# *MTL*–Independent Phenotypic Switching in *Candida tropicalis* and a Dual Role for Wor1 in Regulating Switching and Filamentation

**DOI:** 10.1371/journal.pgen.1003369

**Published:** 2013-03-21

**Authors:** Allison M. Porman, Matthew P. Hirakawa, Stephen K. Jones, Na Wang, Richard J. Bennett

**Affiliations:** Department of Microbiology and Immunology, Brown University, Providence, Rhode Island, United States of America; Duke University Medical Center, United States of America

## Abstract

Phenotypic switching allows for rapid transitions between alternative cell states and is important in pathogenic fungi for colonization and infection of different host niches. In *Candida albicans*, the white-opaque phenotypic switch plays a central role in regulating the program of sexual mating as well as interactions with the mammalian host. White-opaque switching is controlled by genes encoded at the *MTL* (mating-type-like) locus that ensures that only **a** or α cells can switch from the white state to the mating-competent opaque state, while **a**/α cells are refractory to switching. Here, we show that the related pathogen *C. tropicalis* undergoes white-opaque switching in all three cell types (**a**, α, and **a**/α), and thus switching is independent of *MTL* control. We also demonstrate that *C. tropicalis* white cells are themselves mating-competent, albeit at a lower efficiency than opaque cells. Transcriptional profiling of *C. tropicalis* white and opaque cells reveals significant overlap between switch-regulated genes in *MTL* homozygous and *MTL* heterozygous cells, although twice as many genes are white-opaque regulated in **a**/α cells as in **a** cells. In *C. albicans*, the transcription factor Wor1 is the master regulator of the white-opaque switch, and we show that Wor1 also regulates switching in *C. tropicalis*; deletion of *WOR1* locks **a**, α, and **a**/α cells in the white state, while *WOR1* overexpression induces these cells to adopt the opaque state. Furthermore, we show that *WOR1* overexpression promotes both filamentous growth and biofilm formation in *C. tropicalis*, independent of the white-opaque switch. These results demonstrate an expanded role for *C. tropicalis* Wor1, including the regulation of processes necessary for infection of the mammalian host. We discuss these findings in light of the ancestral role of Wor1 as a transcriptional regulator of the transition between yeast form and filamentous growth.

## Author Summary

The white-opaque phenotypic switch has been extensively characterized in the human fungal pathogen *Candida albicans*, where it plays a central role in regulating entry into sexual reproduction. This epigenetic switch is strictly regulated by the *MTL* locus so that only **a** or α cell types can switch to the opaque state, whereas **a**/α cells are locked in the white state. In contrast, we show that in the related pathogen *C. tropicalis* white cells are capable of sexual mating and that the white-opaque switch is independent of *MTL* control. Thus, *MTL* **a**, α, and **a**/α cells all undergo reversible switching between white and opaque states. Despite these differences, switching in both *C. tropicalis* and *C. albicans* is dependent on the expression of the Wor1 transcription factor. This factor is conserved amongst fungal ascomycetes and, in several species, acts as a master regulator of the yeast-to-filament transition. We show that, in addition to regulating the white-opaque switch in *C. tropicalis*, Wor1 expression also promotes filamentation and biofilm formation in this species. We therefore propose that *C. tropicalis* Wor1 has retained the ancestral role of this family of transcription factors while also gaining control over the more recently evolved white-opaque phenotypic switch.

## Introduction

The incidence of opportunistic fungal infections has increased in recent years as a result of immunosuppressive diseases such as AIDS, as well as the use of immunosuppressive drugs in modern medical practices [1]. *Candida* species are typically harmless commensals of humans but are also important fungal pathogens, responsible for both systemic and mucosal opportunistic infections [2]. Most clinically relevant species belong to the *Candida* clade of hemiascomycete yeasts, which diverged from the model yeast *Saccharomyces cerevisiae* 300–600 million years ago [3], [4]. Three *Candida* clade pathogens, *Candida albicans*, *Candida dubliniensis*, and *Candida tropicalis*, have been shown to undergo an epigenetic switch between distinct ‘white’ and ‘opaque’ states [5]–[8], and this phenotypic switch plays a crucial role in modulating behavior. The white-opaque switch has been extensively studied in *C. albicans*, where the two states differ in metabolic preferences [9], environmental responses [10]–[14], interactions with host immune cells [15], [16], and the ability to undergo sexual reproduction [17], [18].

*C. albicans* and *C. dubliniensis* are closely related species that can undergo productive mating with one another [7]. While *C. albicans* represents the most commonly isolated *Candida* species in the clinic, *C. dubliniensis* is rarely found in infections, which may reflect the more limited ability of this species to undergo filamentation [2], [19]. *C. tropicalis* is also a prevalent human pathogen, particularly in individuals with neutropenia or hematologic malignancies [2], and shows a similar overall genome structure to that of *C. albicans*, including synteny at the mating-type-like (*MTL*) locus [20]. *C. albicans* and *C. tropicalis* both contain more than 6,000 protein-coding genes, and analysis of the 5,254 orthologs shared between the two species indicates an average protein sequence identity of ∼70% [20]. However, relatively little is known about the biology of *C. tropicalis* compared to that of the model species *C. albicans*, including the factors that promote pathogenesis in the mammalian host.

In all three *Candida* species, white and opaque forms are distinguished by differences in cell shape, colony morphology, and gene expression profiles. White cells are generally round and give rise to smooth, shiny colonies, while opaque cells are elongated and produce duller, darker colonies [5], [6]. The transcriptional profiles of white and opaque forms are also significantly different, with one-sixth of the transcriptome regulated by the switch in *C. albicans* [9], [21], [22]. Furthermore, white and opaque forms exhibit differences in the propensity to undergo filamentous growth; *C. albicans* white cells are induced to filament in response to multiple environmental stimuli while opaque cells do not generally undergo filamentation [23], [24].

The white-opaque switch plays a particularly prominent role in regulating mating, as only cells in the opaque state undergo efficient conjugation [5], [18]. Switching is regulated by *WOR1* such that loss of this gene prevents formation of the opaque state while, in *C. albicans*, *WOR1* overexpression drives cells into the opaque state [5], [25]–[27]. Genes encoded at the *MTL* locus control white-opaque switching in *C. albicans*; only **a** or α cells switch to the opaque state as a complex between MTL**a**1 and MTLα2 proteins blocks **a**/α cell switching due to repression of *WOR1* [18], [21], [26]. An analysis of 220 clinical isolates of *C. albicans* further demonstrated that only *MTL* homozygous (**a**/**a** or α/α) strains could form stable opaque cells at a detectable frequency [17]. *MTL* regulation ensures that opaque formation only occurs in cells that have the potential to undergo mating, which can take place between *MTL* **a** and *MTL*α cells or via same-sex mating of these cell types [18], [28].

In addition to Wor1, three other transcription factors, Czf1, Wor2, and Efg1, regulate the *C. albicans* white-opaque switch via a network of positive and negative feedback loops [29]. While Wor1 is the master regulator of the opaque state, Czf1 and Wor2 also play positive roles in promoting formation of opaque cells, while Efg1 antagonizes opaque formation and promotes switching to the white state [29]. Surprisingly, the mechanism regulating white-opaque switching in *C. tropicalis* appears distinct from that in *C. albicans*; while switching is dependent on Wor1 in both species, the three associated network transcription factors are not white-opaque regulated in *C. tropicalis* [5]. It is therefore likely that significant differences exist between the mechanisms regulating phenotypic switching in *C. albicans* and *C. tropicalis* [5].

The white-opaque switch is thought to have evolved relatively recently in the *Candida* clade, probably just prior to the divergence of *C. albicans/C. dubliniensis* and *C. tropicalis* [5], [8]. Although this phenotypic switch is limited to within the *Candida* clade, the master transcriptional regulator Wor1 is conserved across the ascomycete lineage [30]. In *S. cerevisiae*, the Wor1 homolog Mit1 acts as part of a transcriptional network to regulate pseudohyphal formation [31], while in the more distantly related ascomycete *Histoplasma capsulatum*, the Wor1 homolog Ryp1 is a master regulator of the transition between yeast and mycelial forms [32]. It has therefore been proposed that the ancestral role of Wor1 was the transcriptional regulation of morphological changes, including the control of filamentous growth [31].

In this study, we investigated the white-opaque transition in *C. tropicalis* and uncovered several features that further distinguish it from the analogous switch in *C. albicans*. We first demonstrate that a stable white-opaque switch can occur in *C. tropicalis* **a**/α cells, indicating that the **a**1/α2 complex does not repress switching in this species. Transcriptional profiling reveals that genes regulated by the white-opaque switch in **a**/α cells show a significant overlap with those regulated by the switch in **a** or α cell types. However, many white-opaque regulated genes are unique to *MTL* heterozygous or *MTL* homozygous cells, indicating that the *MTL* configuration significantly impacts gene expression in the two phenotypic states. Despite these transcriptional differences, the phenotypic switch in all three cell types (**a**, α, and **a**/α) is dependent on the master regulator, Wor1; deletion of Wor1 blocked white-to-opaque switching while overexpression of Wor1 forced cells into the opaque state. We further show that Wor1 promotes filamentous growth in *C. tropicalis*, as elevated *WOR1* expression led to a significant increase in both filamentous growth and biofilm formation. These studies provide new insights into the evolution of Wor1 homologs as conserved regulators of filamentation and epigenetic switching.

## Results

### Comparative Analysis of Mating in *C. tropicalis* White and Opaque Cells

A key feature of the *C. albicans* white-opaque switch is that **a**/α cells are locked in the white state due to repression of *WOR1* transcription by the **a**1/α2 heterodimer. A direct consequence of this control is that **a**/α mating products formed between opaque **a** and α cells typically form white colonies [18]. Rare opaque colonies (<5%) were observed in *C. albicans* mating products, but in each case these colonies had undergone loss of **a**1 or α2, thereby relieving repression of *WOR1* and allowing propagation of the opaque state [18]. In *C. tropicalis*, the white-opaque switch also regulates mating, although the difference in mating efficiency between white and opaque forms is less striking than that in *C. albicans*. Whereas *C. albicans* opaque cells mate a million times more efficiently than white cells [18], mating of *C. tropicalis* opaque cells is only about a hundred times greater than that of white cells [5].

**Figure 1:**
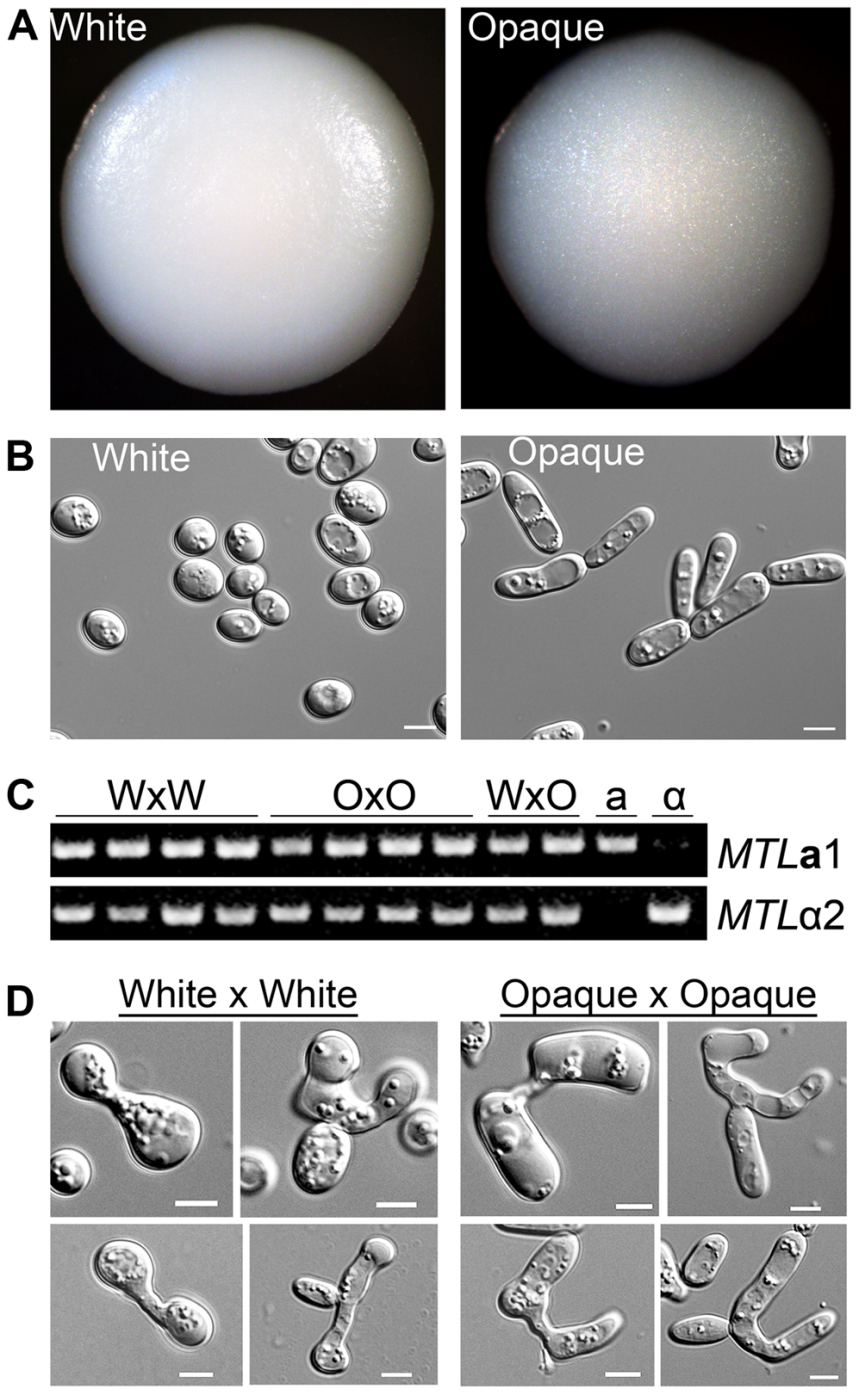
Products of *C. tropicalis* white×white and opaque×opaque mating maintain parental phenotypes. (A) Colony morphology of mating products. Left, product of mating *C. tropicalis* white **a** (CAY3376) with white α (CAY3391) cells. Right, product of mating opaque **a** (CAY3378) with opaque α (CAY3392) cells. Colonies were grown on SCD medium lacking histidine and arginine for 3 days at room temperature to select for mating products. (B) Cell morphology of cells taken from the corresponding colonies. Scale bars = 5 µm. (C) PCR to verify the presence of *MTL* **a**1 and *MTL*α2 genes in white×white (WxW) mating products, opaque×opaque (OxO) mating products, white×opaque (WxO) mating products, and in the parental **a** and α strains. (D) Left, pictures of zygotes analyzed from white×white mating experiments and, right, pictures of zygotes analyzed from opaque×opaque mating experiments. Scale bars = 5 µm.

These observations led us to hypothesize that *C. tropicalis* white cells may be capable of appreciable mating frequencies, even without switching to the opaque state. Close inspection of *C. tropicalis* **a**/α mating products revealed that colonies generated by mating white **a** and α cells were distinct from those formed by mating opaque cells. This is illustrated in Figure 1A, where colonies formed from mating between white **a** and α cells exhibited a shiny, smooth appearance, while colonies formed by products of mating between opaque cells were consistently duller and darker. Examination of the cells from these colonies revealed that the products of white×white mating were round, resembling classical white cells, while the products of opaque × opaque mating were elongated, reminiscent of opaque cells (Figure 1B). PCR analysis of the mating products confirmed that **a**1 and α2 were still present in the products formed from mating white or opaque cells (Figure 1C). The **a**1 and α2 loci were also sequenced and shown to exactly match the published gene sequences [20], indicating that these genes were not mutated in the *C. tropicalis* strains used for these experiments. Together, these results indicate that the *C. tropicalis* white-opaque switch is not under strict regulation by the *MTL* locus. Thus, unlike *C. albicans*, mating of *C. tropicalis* opaque **a** and α cells (and formation of the **a**1/α2 complex) does not force cells back to the white form. Instead, *C. tropicalis* **a**/α mating products inherit the phenotype of their parental cells and can even stably propagate in the opaque form.

These experiments reveal another important aspect of *C. tropicalis* biology, as they establish that white cells can mate without switching to the opaque form. Our observation that the products of white and opaque cell mating are distinguishable, and that white×white mating generates white mating products, indicates that *C. tropicalis* white cells can directly undergo conjugation with one another. In support of the fact that both white and opaque cells can mate, we also observed structural differences in zygotes formed between white cells and those formed between opaque cells. Zygotes formed from white cells were generally smaller and contained rounder cells than zygotes formed from opaque cells (Figure 1D). We conclude that *C. tropicalis* white cells are competent for mating, albeit at a lower efficiency than that of opaque cells.

To complete this analysis, we also examined the products of mating in crosses between white and opaque cells. Interestingly, the products of these mixed crosses were predominantly opaque (data not shown). Since Wor1 expression is essential for induction of the opaque form, we surmise that Wor1 protein from the parental opaque cell is present at sufficient levels in the zygote to result in stable propagation of the opaque state in these mating products.

### Phenotypic Switching in *C. tropicalis* **a**/α Cells

**Figure 2:**
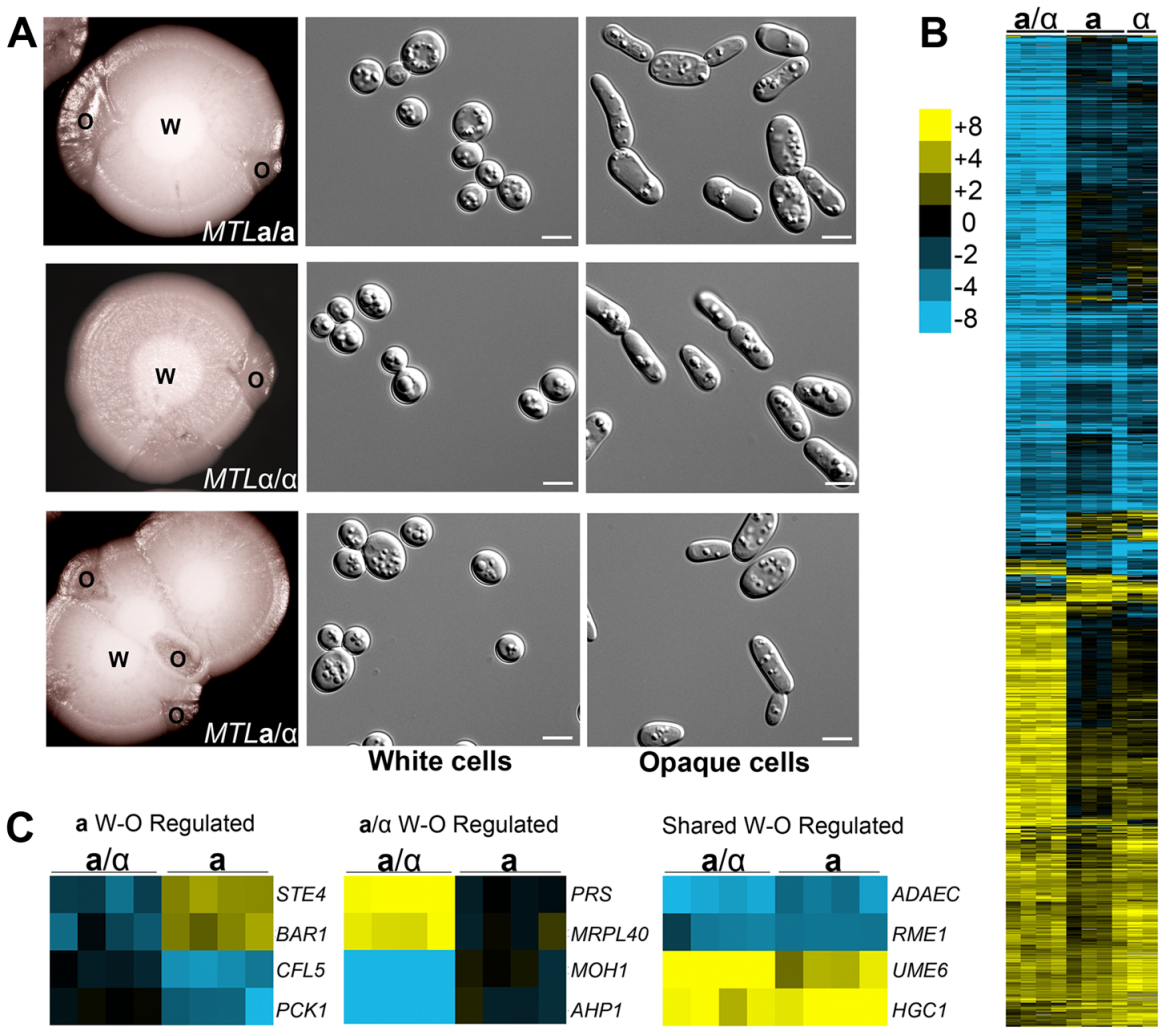
*C. tropicalis* a/α cells undergo a white-opaque phenotypic switch related to that in a and α cells. (A) White (W) colonies sectoring to opaque (O) in **a** (CAY1503), α (CAY1505), and **a**/α (CAY1511) strains and the corresponding cell morphologies observed from these colonies. Cells were grown in SCD at 37°C for 5 days and plated to Lee's + N-acetylglucosamine at room temperature for 10 days. Scale bars = 5 µm. (B) Opaque-white gene expression profiles of **a**/α, **a**, and α cells. cDNA prepared from white and opaque states of CAY1511 (**a**/α), CAY1504 (**a**), and CAY1505 (α) in independent experiments was hybridized against a universal reference. Opaque cell gene expression was divided by that in white cells and filtered for genes with a fold-change greater than 4 and clustered by Average Linkage Clustering. Genes with elevated expression in white cells are shown in blue and genes upregulated in opaque cells are shown in yellow. (C) Examples of genes whose expression was white-opaque regulated in **a** cells (left), **a**/α cells (middle), or in both **a** and **a**/α cells (right).

A previous study by Xie *et al*. indicated that **a**/α cells of *C. tropicalis* were able to form opaque cells based on similar cell morphologies to **a**/**a** and α/α opaque cell types [8]. To address whether **a**/α cells could directly switch to the opaque state, cells were grown on medium containing N-acetylglucosamine, as this was previously shown to induce *C. tropicalis* switching [8]. Rare colony switching was observed with darker sectors forming at the edge of **a**/α colonies, similar to that in conventional white-to-opaque sectoring of **a** and α colonies (Figure 2A). Cells from the **a**/α sectors were elongated rather than spherical, a characteristic feature of opaque cells [5]. Additionally, white **a**/α cells had a smooth surface while opaque **a**/α cells had an uneven or pimpled surface, and thus resembled conventional white and opaque cells (Figure S1A and [33]). Once formed, diploid opaque **a**/α cells were stably maintained in the opaque state, similar to tetraploid opaque **a**/α mating products (Figure 1A and data not shown). The frequency of white-to-opaque switching in **a**/**a** and **a**/α cells varied widely from experiment to experiment (0–7% switching), but the difference in switching between strains was not statistically significant.

As the **a**1/α2 complex acts to repress white-to-opaque switching in *C. albicans*, we also examined whether the **a**1 and α2 genes were expressed in *C. tropicalis* opaque **a**/α cells. Reverse transcription (RT)-PCR was performed on each cell type and revealed that **a**1 and α2 genes were actively expressed in **a**/α cells regardless of phenotype (Figure S1B). This result indicates that the white-to-opaque switch in *C. tropicalis* appears independent of **a**1 and α2 function.

We further examined 8 additional *C. tropicalis* clinical **a**/α strains for their ability to undergo the white-opaque switch. Opaque formation was observed in 2 of these strains, indicating that other **a**/α strains are able to undergo the white-opaque switch (Table S1). The presence of the **a**1 and α2 genes in switching strains was confirmed by PCR. These genes were sequenced from opaque cells derived from one of the three clinical **a**/α strains and also found to match the published sequence (data not shown). Our results therefore establish that *C. tropicalis* white-opaque switching occurs independent of *MTL* control and that multiple **a**/α strains can switch to a stable opaque state.

### Transcriptional Profiling of *C. tropicalis* White and Opaque States

To ascertain the relationship between genes regulated by the white-opaque switch in *MTL* heterozygous **a**/α cells with those in *MTL* homozygous **a**/**a** or α/α cells, transcriptional profiling was performed on cells from both morphological states in each cell type (Figure 2B). SAM (Statistical Analysis of Microarrays) was used to determine gene expression changes that were significant for white-opaque regulated genes in **a** and **a**/α cells (see Materials and Methods and Tables S5 and S6). Expression profiles of **a**/α white and opaque forms showed significant overlap with the corresponding white- and opaque-specific genes in **a** cells (Figure 2B). In particular, of the 120 genes significantly upregulated in white **a** cells, 73 of these genes were also upregulated in white **a**/α cells, while of the 129 genes upregulated in opaque **a** cells, 22 of these genes were also upregulated in opaque **a**/α cells (Figure S2). These results establish that *C. tropicalis* **a**/α cells undergo a white-opaque switch related to that in *MTL* homozygous cells [5], and that there is significant overlap between white-opaque genes regulated in *MTL* homozygous and heterozygous cell types (p<1e-150 and p<1e-20 for white- and opaque-specific genes, respectively).

Despite significant overlap between white-opaque regulated genes in the different cell types, a large number of switch-regulated genes were unique to either *MTL* heterozygous or *MTL* homozygous cells. This was particularly striking in **a**/α cells, where the expression of 549 genes was significantly different between white and opaque forms, while only 249 genes were white-opaque regulated in **a** cells (Figure 2B, 2C and Figure S2). Many of the additional white-opaque regulated genes in **a**/α cells were involved in translation, biosynthetic processes, or gene expression based on GO term analysis (Text S1 and Figure S3). This indicates that the switch in **a**/α cells regulates additional cellular processes to those in **a** or α cells, and these differences could have important implications for the role of the switch in *MTL* heterozygous strains.

Conversely, white and opaque **a** and α cells also differentially expressed genes that were not white-opaque regulated in **a**/α cells. For example, the mating-related genes *STE4* and *BAR1* were expressed at higher levels in opaque *MTL* homozygous cells than in white cells, but were not differentially expressed between white and opaque states in *MTL* heterozygous cells (Figure 2C). Taken together, these results indicate that *MTL* homozygous and *MTL* heterozygous cells share related white-opaque expression profiles, but that the genes regulated by this switch are also influenced by the configuration of the *MTL* locus.

### Mating of *C. tropicalis* **a**/α Cell Types

**Figure 3:**
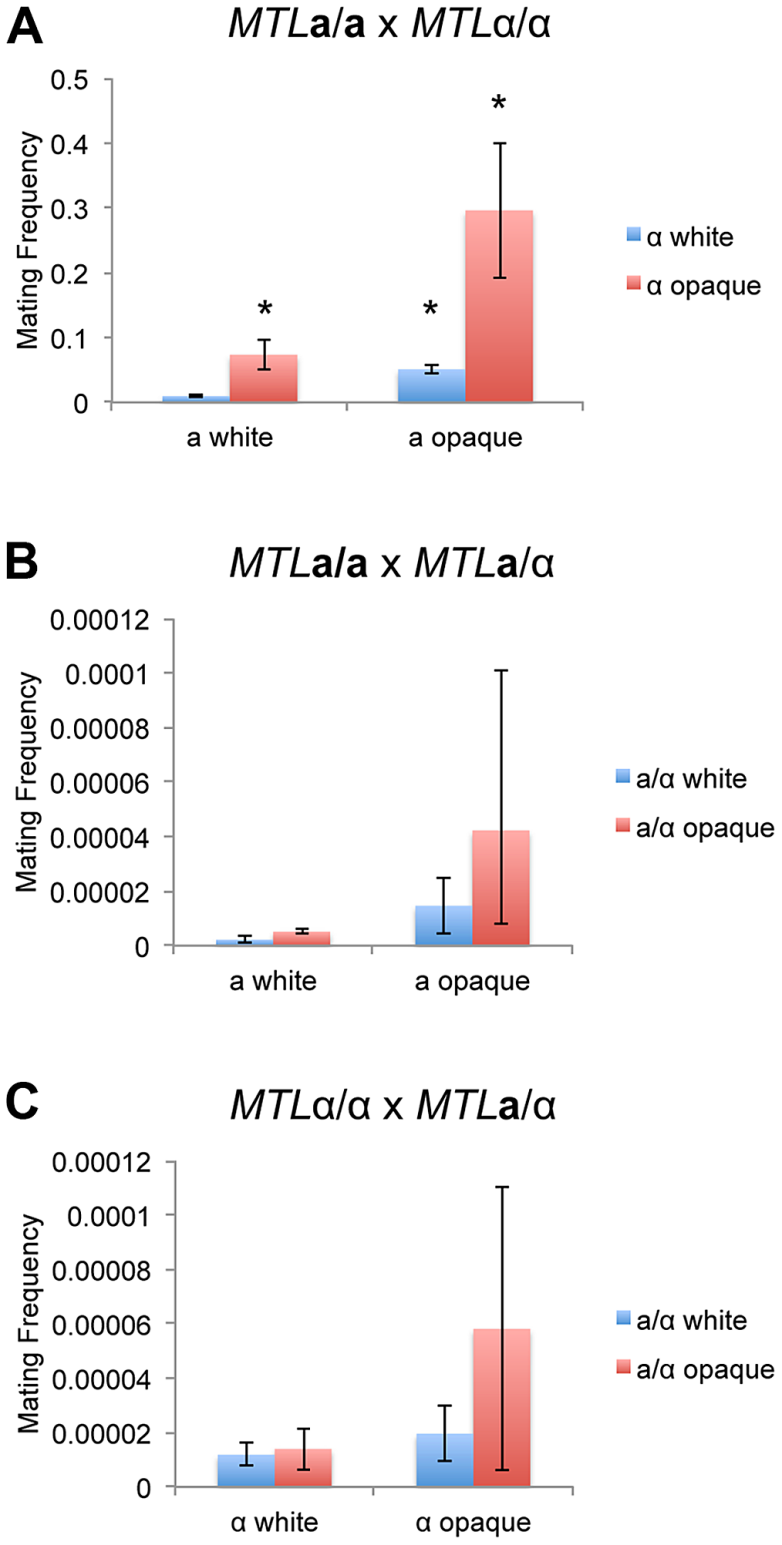
The white-opaque switch regulates *C. tropicalis* mating in a, α, and a/α cells. Mating frequency of (A) **a** x α (CAY1503 x CAY1505), (B) **a** x **a**/α (CAY1503 x CAY1511), and (C) α x **a**/α (CAY1505 x CAY1513) white and opaque cells. Mating experiments were performed on Spider medium for 3 days at room temperature and plated to selective media to quantify mating frequency (see Materials and Methods). Error bars indicate SEM for 3 independent experiments. * = significantly different from white×white crosses, p<0.01.

A key role of the white-opaque switch in *Candida* species is the regulation of sexual reproduction [5], [18]. Given that *C. tropicalis* **a**/α cells undergo the white-to-opaque switch, we tested whether these cell types undergo productive mating. We found that **a**/α cells could not mate with other **a**/α cells, and that same-sex mating of **a** or α cells was also not observed (data not shown), in agreement with previous studies [5]. However, low levels of mating were obtained between **a**/α cells and either **a** or α cells as partners. The frequency of this **a**/α cell mating was 10^3^ to 10^5^-fold lower than conventional mating between **a** and α cells (Figure 3 and Figure S4). Mating could be detected using both white and opaque **a**/α cells, and although **a**/α cell mating was higher in the opaque state, this difference was not significant.

*C. albicans* **a**/α cells from the SC5314 background do not undergo mating with other cell types ([18] and data not shown). However, overexpression of *WOR1* in these cell types can override **a**/α control, forcing them to adopt the opaque state and promoting low frequency mating with **a** or α cells [25]. These studies indicated that productive mating was likely due to loss of *MTL* genes, allowing *C. albicans* **a**/α cells to mate as **a** or α cells [25]. We similarly suggest that low level mating of *C. tropicalis* **a**/α cells is due to *MTL* instability, as loss of *MTL* genes was frequently detected in the mating products from **a**/α crosses (Text S1, Figure S5, Table S11). In addition, genetic recombination at the *MTL* was observed in a subset of mating products, indicating that homozygosis of the *MTL* could promote **a**/α mating (Figure S5). However, regardless of the mechanism involved, mating of **a**/α cells occurred at a very low frequency compared to conventional mating between **a** and α cells.

### Transcriptional Regulation of *C. tropicalis* Phenotypic Switching

**Figure 4:**
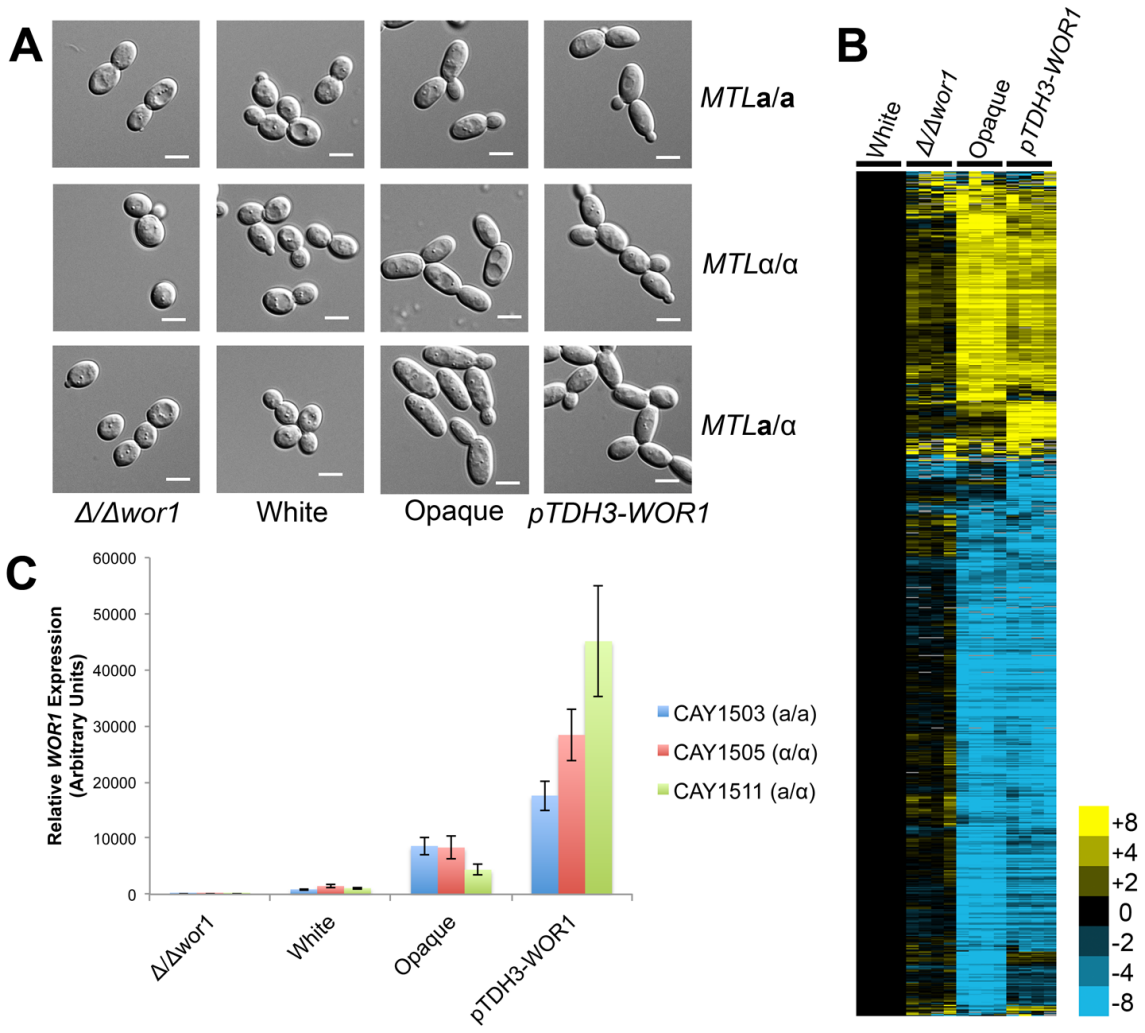
*WOR1* is the master regulator of the white-opaque switch in *C. tropicalis*. (A) Cell morphologies of *Δwor1*, white, opaque, and *pTDH3-WOR1* (*WOR1*-overexpressing) strains. Cells were grown in Spider medium at room temperature to 0.8–1.0 OD_600_. Scale bars = 5 µm. (B) Gene expression in **a**/α white cells (CAY1511), *Δwor1* cells (CAY4043), opaque cells (CAY4048), and *pTDH3-WOR1* cells (CAY4045), relative to white cells (CAY1511). Expression profiles for each state were divided by white expression values and filtered for those genes with an expression change greater than 4-fold in 4 or more experiments. (C) *WOR1* expression in *Δwor1*, white, opaque, and *pTDH3-WOR1* strains derived from **a**, α, and **a**/α cells. Expression levels measured by qRT-PCR. Error bars indicate SEM for replicate experiments from 3 different biological replicates.

*WOR1* is the master transcriptional regulator of the white-opaque switch in both *C. tropicalis* [5] and *C. albicans* [25]–[27], and its expression is therefore critical for opaque cell formation. To further investigate the role of *WOR1*, we constructed *Δwor1/Δwor1* and *WOR1* overexpression strains in *C. tropicalis* **a**, α, and **a**/α backgrounds. Initially, a *pACT1-WOR1* construct was used to overexpress *WOR1*. However, unlike in *C. albicans*, this construct failed to induce switching to opaque in *C. tropicalis* (data not shown and [8]). Instead, an alternative promoter, *TDH3*, was utilized for *WOR1* expression and found to be sufficient to induce opaque formation in *C. tropicalis* **a**, α, and **a**/α cells (Figure 4A). Thus, *WOR1* expression is both necessary and sufficient for formation of the opaque state in *C. tropicalis*.

Previously, we showed that *wor1* mutant **a** or α cells do not undergo detectable mating in *C. tropicalis* [5]. Mating assays were also performed on *WOR1* overexpression strains and these strains exhibited increased mating between **a** and α cells (∼70% mating) relative to wild-type opaque strains (∼15% mating, Figure S4). This result further establishes the key role of Wor1 in directing expression of genes necessary for efficient conjugation.

Transcriptional profiling was also performed on *wor1* mutant and *WOR1* overexpression **a**/α strains, and profiles compared to wild-type white and opaque cells (Figure 4B). Consistent with the key role of *WOR1* in directing the white-opaque switch, the expression profile of *Δwor1* strains was similar to that of wild-type white cells, while strains overexpressing *WOR1* had an expression profile similar to that of wild-type opaque cells (Figure 4B). However, although the global expression patterns were similar, many genes were differentially expressed between *Δwor1* strains and white cells (Tables S7 and S8) and between *WOR1* overexpression strains and opaque cells (Tables S9 and S10). Expression of *WOR1* was also compared by RT-PCR and was found to have the following relative transcription levels: *WOR1* overexpresser > wild-type opaque > wild-type white > *wor1* mutant (Figure 4C). *WOR1* expression was ∼2–8-fold higher in *WOR1* overexpression strains relative to opaque strains, ∼4–10-fold higher in opaque cells relative to white cells, and ∼1000-fold higher in white cells relative to *wor1* mutants. The difference in expression levels between white cells and *wor1* mutants is consistent with low-level expression of this gene in the white state. We also note that *WOR1* expression levels were in line with the relative mating efficiencies of these strains (Figure S4).

In *C. albicans*, the white-opaque switch is regulated by Wor1 together with three other factors, Wor2, Czf1, and Efg1, as part of an interacting transcriptional circuit [29]. The *EFG1* gene is missing from the *C. tropicalis* genome although a gene encoding a related APSES transcription factor, *EFH1*, is present [20]. Previous studies failed to observe differential expression of *WOR2*, *CZF1*, or *EFH1* between *C. tropicalis* white and opaque **a** cells [5]. We examined the expression of these genes in the larger white-opaque data set present in **a**/α cells, and between *wor1* mutant and *WOR1* overexpression **a**/α strains. *CZF1* and *EFH1* did not show significant expression differences between these profiles, while *WOR2* expression was decreased in opaque cells, the opposite of that expected based on its expression in *C. albicans.* These results are therefore consistent with transcriptional control of the white-opaque switch being divergent between *C. tropicalis* and *C. albicans* (outside of the conserved role of Wor1).

We also identified several other transcription factors regulated by the *C. tropicalis* white-opaque switch. Among these factors, *UME6*, a gene known to have a role in regulation of filamentous growth in *C. albicans*, had consistently higher expression in *C. tropicalis* opaque cells. Conversely, upregulated genes in white cells included *RME1* and *NDT802*, genes with homologs involved in regulating meiosis in *S. cerevisiae*, as well as *ADAEC*, a gene that is also white-specific in *C. albicans*. Further studies are now required to determine if any of these transcription factors play an active role in regulating the white-opaque switch in *C. tropicalis.*

### Wor1 Regulates Filamentation and Biofilm Formation in *C. tropicalis*

**Figure 5:**
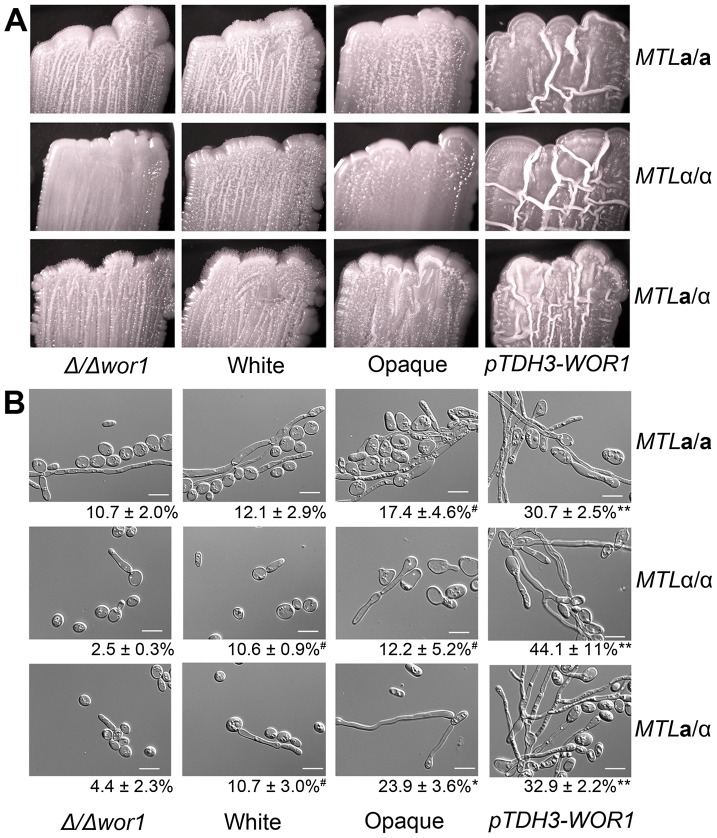
*C. tropicalis WOR1* expression regulates filamentous growth. (A) Wild-type white, wild-type opaque, *αwor1* mutant, and *WOR1*-overexpression (*pTDH3-WOR1*) strains were grown on Spider medium at 37°C for 7 days and photographed. (B) Cells from corresponding patches in (A). The percentage of filamentous cells is shown below each image. Scale bar = 10 µm. ** = significantly different from opaque, white, and *Δwor1* strains, p<0.001. * = significantly different from white and *Δwor1*, p<0.001. ^#^ = significantly different from *Δwor1*, p<0.05.

In several diverse fungal species, Wor1 homologs do not mediate white-opaque phenotypic switching but still act as transcriptional regulators of cellular morphogenesis. This is evident in *S. cerevisiae* and *H. capsulatum*, where Wor1 homologs regulate the transition from budding yeast forms to filamentous forms [31], [32]. Colony morphologies of *C. tropicalis* strains were compared on several media, and it was found that *WOR1* overexpression strains were markedly more wrinkled than wild-type white or opaque cells when grown on Spider medium (Figure 5A). Spider medium is a low nutrient medium that also induces filamentation in *C. albicans* [34]. Examination of cells from the wrinkled colonies confirmed that Wor1 overexpression strains were significantly more filamentous than other cell types due to a higher percentage of both hyphal and pseudohyphal cells (Figure 5B). In addition, filamentation generally increased as *WOR1* gene expression levels increased. Thus, for **a**/α cells, filamentation increased from *wor1* mutants (4.4%) to white cells (10.7%) to opaque cells (23.9%) to *WOR1* overexpressing cells (32.9%, Figure 5B). These results indicate that filamentation correlates with *WOR1* expression in *C. tropicalis*, and that Wor1 regulates filamentous growth independent of its regulation of the white-opaque switch.

**Figure 6:**
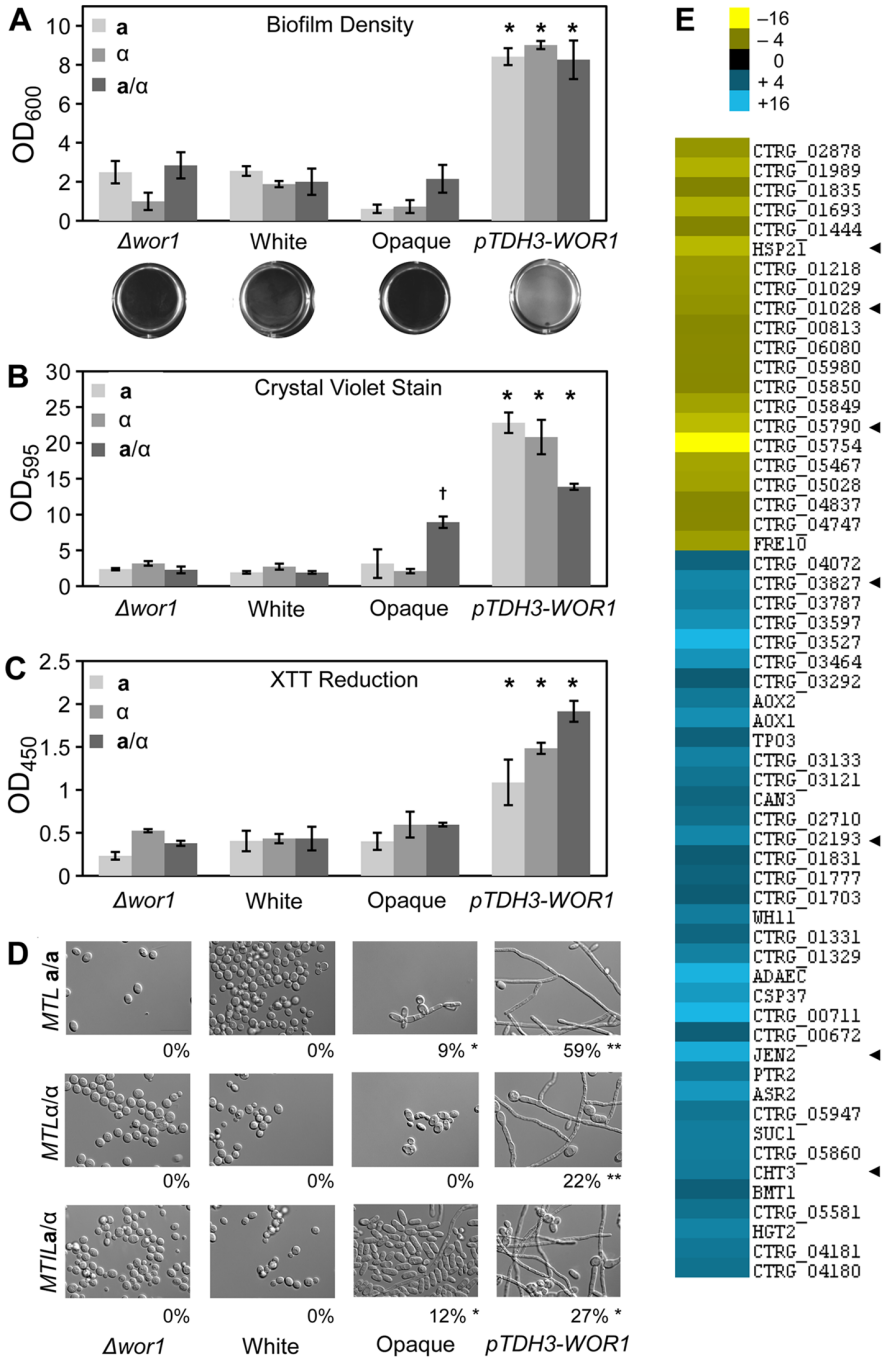
Biofilm formation is induced by *WOR1* overexpression in *C. tropicalis*. (A) Quantification of cells adhering to a 12-well polystyrene dish following a 48-hour incubation in Lee's medium. Representative images of wells appear below the corresponding strains. Data from three experimental replicates, error bars indicate SD. * = p<0.001 when compared to *Δwor1*, white, and opaque strains. (B, C) Quantification of biofilm production by colorometric assays. (B) Beta-glucan content was determined by crystal violet staining. (C) Cellular viability was measured by formazan formation upon XTT reduction. Each biofilm experiment includes three or more replicates, error bars indicate S.D. * = p<0.001 when compared to *Δwor1*, white, and opaque strains. † = p<0.001 when compared to *Δwor1*, white, and *pTDH3-WOR1* strains. (D) Morphology of adherent cells taken from (A). Percentage of filamentous cells indicated below image. Scale bars = 20 µm. Statistical significance was determined using Mann-Whitney pair-wise tests. ** = significantly different from opaque, white, and *αwor1* strains, p<0.001. * = significantly different from white and *αwor1* strains, p<0.001. (E) Genes induced in *MTL* **a**/**a** *WOR1* overexpressing cells under biofilm conditions. Heat map shows relative expression changes between *pTDH3-WOR1* (CAY3853) and opaque (CAY3378) strains in the adherence to plastic biofilm assay. Data set was filtered for genes with a fold-change greater than 4 and clustered by Average Linkage Clustering. Data shows average of two independent biological replicates. Arrowheads highlight *C. albicans* homologs that are biofilm regulated.

Filamentous growth is directly associated with biofilm formation in *C. albicans*. Biofilms are surface-associated communities of cells and often involve both yeast and filamentous cells in stable, complex structures that form on biotic and abiotic surfaces [35], [36]. Furthermore, *C. albicans* biofilms are responsible for the seeding of serious bloodstream infections and are associated with antifungal drug resistance [35], [36]. We therefore examined whether *C. tropicalis wor1* mutant cells, white cells, opaque cells, or cells overexpressing *WOR1* displayed differences in their ability to form biofilms using an adherence to plastic assay. White cells, opaque cells, and *wor1* mutants generally performed poorly in these adherence assays, regardless of *MTL* configuration (Figure 6A). In contrast, cells overexpressing *WOR1* generated robust biofilms on the polystyrene plates, as noted by visual inspection as well as quantification of the biofilm by optical density (Figure 6A). Increased biofilm formation was also demonstrated by an elevated level of staining with crystal violet, which indicates extracellular matrix production (Figure 6B), and by increased XTT reduction, indicating that there were significantly more adherent, viable cells in the *WOR1* overexpressing biofilms (Figure 6C). Many cells within the *WOR1*-overexpressing biofilms were filamentous (22–59%), whereas comparatively few cells were filamentous in white, opaque, or *Δwor1* cells (0–12%, Figure 6D).

Transcriptional profiling was performed under biofilm culture conditions on *WOR1* overexpression strains and compared to gene expression in wild-type opaque strains. This revealed that 58 genes were differentially expressed (>4-fold) between these strains (Figure 6E). Gene expression changes included *CHT3*, which encodes a chitinase whose expression is repressed in *C. albicans* hyphal cells [37], and which was also repressed in the *C. tropicalis WOR1* overexpression strain. Conversely, *JEN2* was upregulated in *C. tropicalis WOR1*-mediated biofilms, and encodes a dicarboxylic acid transporter whose expression is also upregulated in *C. albicans* biofilms [38]. In addition, several *C. tropicalis* white-specific genes (e.g., *ADAEC* and *WH11*) were further downregulated in the *WOR1*-overexpressing cells relative to opaque cells (Figure 6E). However, no genes with defined roles in filament regulation were obtained from these profiling studies, although many of the differentially regulated genes currently have no known function.

Together, these results demonstrate that Wor1 promotes filamentous growth and biofilm formation in *C. tropicalis* and these functions are independent of the white-opaque switch. The role of Wor1 in this species is therefore analogous to that of several distantly related Wor1 homologs in the fungal lineage. The increase in filamentous growth is presumably a major reason for the increased adhesion and biofilm formation in the *C. tropicalis* Wor1-overexpression strains.

### Contribution of *MTL* Configuration to *C. tropicalis* Fitness

We next determined if the configuration of the *MTL* locus contributes to overall fitness in *C. tropicalis*. In particular, we investigated the possibility that **a**/α cells exhibit increased fitness over *MTL* homozygous cells. We first analyzed 150 natural *C. tropicalis* isolates (Table S1) to determine their *MTL* configuration and found that 119 isolates (∼80%) were **a**/α strains, while 23 (∼15%) were **a** strains and 8 (∼5%) were α strains. A similar analysis performed by Xie *et al*. showed an even higher prevalence of **a**/α strains (145/150 isolates) in the natural population [8]. Thus, similar to the case in *C. albicans* [17], [39], **a**/α genotypes are the predominant cell types in natural *C. tropicalis* isolates.

We speculated that the predominance of **a**/α cells could result from increased fitness of these cell types relative to **a** or αcells. Thus, the ability to switch as an **a**/α cell could allow cells to retain their optimal fitness yet also be competent to undergo the white-opaque switch. The fitness of *C. tropicalis* **a**, α, and **a**/α strains was addressed using an *in vivo* model of murine candidiasis. Competition experiments were performed between **a**/α and **a** cells, or between **a**/α and α cells, using strains carrying different auxotrophic markers. Neutropenia was induced prior to infection with *C. tropicalis* cells as this increases the fungal load associated with systemic disease [40]. Mixtures of strains were injected into the tail veins of neutropenic mice and cells recovered from the brain and kidneys 72 hours following infection and characterized for cell type.

**Figure 7:**
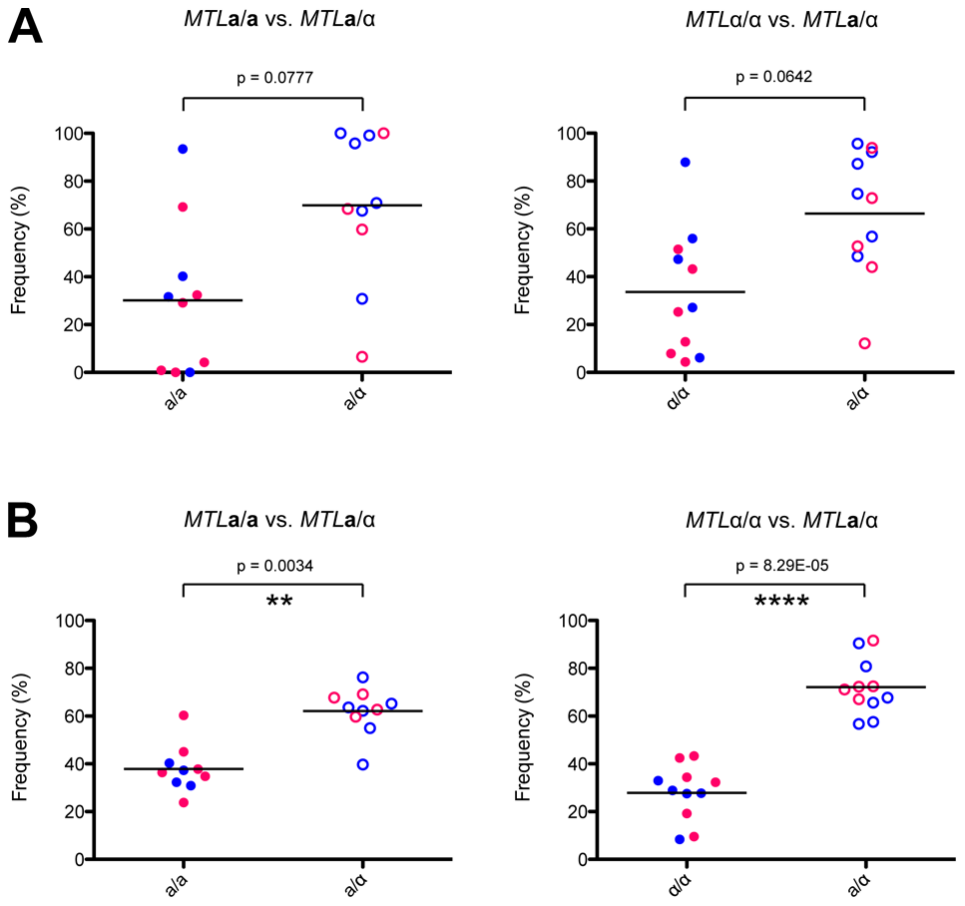
Heterozygosity at the *MTL* provides a fitness advantage *in vivo*. *MTL* homozygous and heterozygous *C. tropicalis* cells were mixed 1∶1 and injected systemically via the mouse tail vein. After 72 hours, *C. tropicalis* cells were recovered from the kidney (A) and the brain (B), and strain genotypes identified by plating to auxotrophic media. In both organs, **a**/α cells (CAY1511 or CAY1513) showed increased fitness relative to **a** (CAY1502 or CAY1503) orα (CAY1505 or CAY1509) cells, and this difference was statistically significant in the brain. ** = p<0.01, **** = p<0.0001. Blue circles indicate strains were His-, red circles indicate strains were Arg-.

Regardless of the combination of auxotrophic markers used, **a**/α cells consistently colonized host organs at a higher fungal burden than **a** or α cells in competition assays (Figure 7). This difference was statistically significant for fungal colonization of the brain, where approximately 70% of the recovered cells were **a**/α cell types. These experiments indicate that **a**/α cells are fitter than **a** or α cells in this *in vivo* model, as they outcompeted *MTL* homozygous cell types during systemic infection. We propose that phenotypic switching in **a**/α cells allows these cells to have the potential to adopt both white and opaque forms while still maintaining their fitness advantage over **a** or α strains.

## Discussion

In this study, we show that a Wor1-regulated phenotypic switch operates in *C. tropicalis* **a**, α, and **a**/α cell types and is therefore independent of *MTL* control. Even in strains expressing intact **a**1 and α2 genes, white cells could stably switch to opaque cells, indicating that *MTL* genes do not block switching. The regulation of the white-opaque switch in *C. tropicalis* therefore deviates from that in *C. albicans*, where the action of **a**1/α2 prohibits efficient opaque formation in **a**/α cells [18]. The discovery that *C. tropicalis* **a**/α cells can undergo stable formation of the opaque state raises new questions as to the role(s) of the switch *in vivo*, as well as the transcriptional regulation of the switch in this species. We note that Xie *et al*. also recently observed a white-opaque-like morphological switch in two *C. tropicalis* **a**/α strains [8], and although the integrity of **a**1 and α2 genes was not reported, it is likely that these strains were also capable of *MTL*-independent white-opaque switching.

### *C. tropicalis* Mating and the White-Opaque Switch

Despite its apparent independence from *MTL* control, the *C. tropicalis* white-opaque switch still regulates sexual mating in this species. Mating between *C. tropicalis* **a** and α opaque cells occurs approximately 100 times more efficiently than that between white cells. It was not known, however, if mating of white cells required that cells first switch to the opaque state prior to mating, or if white cells were themselves mating competent. Analysis of *C. tropicalis* white×white and opaque×opaque mating products addressed this question, as they revealed that mating products inherited the phenotypic state of the parental cells (i.e. mating between white cells generated white mating products while those between opaque cells generated opaque mating products). This simple observation established two important facts about phenotypic switching and mating in *C. tropicalis*. First, it demonstrated that the *C. tropicalis* white-opaque switch is independent of *MTL* status, as **a**/α cells formed by mating would be expected to be in the white state, regardless of the phenotype of the parental cells. Indeed, this is what has been observed in *C. albicans*, where the products of mating are white or, if opaque, have undergone concomitant loss of *MTL* genes [18]. Second, it showed that *C. tropicalis* white cells are themselves mating competent; if they had switched to opaque prior to mating then the products of mating would also have been opaque. Thus, *C. tropicalis* white and opaque cells are both mating competent, although the efficiency of mating is higher in the opaque state than in the white state.

The discovery that *C. tropicalis* white cells are mating competent is interesting given that mating is completely abolished in mutant cells lacking *WOR1*. Thus, although *C. tropicalis* white cells mate inefficiently, their mating frequency is still more than 1000-fold higher than that of *wor1* mutants [5]. This indicates that significant *WOR1* expression occurs in *C. tropicalis* white cells (supported by array and qPCR data), and that this expression is sufficient for basal induction of genes necessary for conjugation. In opaque cells, Wor1 levels are further increased over white cells (4–10 fold) and higher mating efficiencies are observed in cells with the opaque phenotype. Candidate mating genes include *STE4* and *BAR1*, both of which are elevated in the opaque state and are known to regulate mating in diverse fungal species [41]–[43]. Mating efficiency was further increased in Wor1 overexpression strains compared to wild-type opaque cells. These results demonstrate that mating efficiency can be separated from the white-opaque switch *per se* and, at least to a first approximation, *C. tropicalis* mating correlates directly with Wor1 expression levels.

Mating was also observed between *C. tropicalis* **a**/α cells and either **a** or α partners. The efficiency of **a**/α cell mating was very low (∼10^−6^) regardless of whether white or opaque cells were used, and was approximately 5 orders of magnitude lower than conventional mating between opaque **a** and α cells. In fact, mating of **a**/α cells was 1000-fold lower than that between white **a** and α cells. Furthermore, the majority of **a**/α mating products had lost *MTL* genes or had undergone recombination at the *MTL*, and therefore had presumably mated as **a** or α cell types. Aberrant mating of **a**/α cells has also been described in *C. albicans* cells overexpressing *WOR1* and was also attributed to rare loss of *MTL* genes [25]. Moreover, it is known that *C. albicans* **a**/α cells undergo occasional mating upon loss of **a**1 or α2 genes, as **a**1/α2 represses the expression of haploid-specific genes necessary for mating [18], [21].

### Profiling of *C. tropicalis* Phenotypic States

Transcriptional profiling of the three different genotypes (**a**, α, and **a**/α) in *C. tropicalis* revealed that there was significant overlap between white-opaque regulated genes in each cell type. This is to be expected given that Wor1 regulates the switch irrespective of *MTL* configuration. The key role of this transcription factor in determining the phenotypic state was further illustrated by comparing the profiles of white cells, opaque cells, *wor1* mutants, and *WOR1* overexpression strains. The profiles of white cells and *wor1* mutant cells were similar, as were those of opaque cells and *WOR1* overexpressing cells. In addition, *wor1* mutant strains did not undergo switching to opaque, while *WOR1* overexpression locked cells in the opaque state. Together, these results establish Wor1 as the master regulator of the *C. tropicalis* white-opaque switch in **a**/α cells, similar to its role in **a** and α cells.

Surprisingly, significant differences in white-opaque regulated genes were noted between *MTL* homozygous and *MTL* heterozygous strains. For example, *STE4* and *BAR1* were expressed at elevated levels in opaque **a** or α cells, but not in opaque **a**/α cells. This may reflect the fact that the white-opaque switch regulates mating between **a** and α cells, but does not significantly promote mating in **a**/α cells. In addition, ∼400 switch-regulated genes were unique to **a**/α cells and were not observed in **a** or α cell types. The gene set unique to opaque **a**/α cells included a significant association with translation, biosynthetic processes, and gene expression control. A direct implication of these transcriptional differences is that behavioral differences (including effects on host interactions and virulence) may occur between white and opaque cells from *MTL* homozygous and *MTL* heterozygous cell types, and additional experiments will now test this possibility.

### Evolution and Function of Phenotypic Switching in *Candida* Species

The white-opaque switch affects virtually every aspect of *Candida* biology, from mating to interactions with host immune cells to pathogenesis [44]–[47]. *MTL* regulation of the switch in *C. albicans* ensures that only those cells that are capable of mating undergo the switch to the mating-competent form [18]. The discovery that *C. tropicalis* can form opaque cells regardless of *MTL* status suggests that the switch may have originally evolved to regulate processes other than sexual mating. Furthermore, the majority of natural *C. tropicalis* and *C. albicans* isolates are **a**/α strains (80–95%); thus, while most *C. albicans* strains are unable to switch, it appears that the majority of *C. tropicalis* isolates are competent for switching to the opaque form.

Many of the genes controlled by the white-opaque switch in *C. albicans* are metabolism genes, perhaps reflective of the fact that white and opaque cells colonize different host niches [9], [21]. *C. albicans* white cells exhibit greater virulence in models of systemic infection, while opaque cells are more efficient at colonization of the skin and are unstable at 37°C, rapidly switching back to the white form [48], [49]. In contrast to *C. albicans*, *C. tropicalis* opaque cells are stable at 37°C [5] and may colonize host niches that are refractory to *C. albicans* opaque cells. In addition, we found that *C. tropicalis* **a**/α cells exhibited increased fitness relative to **a** or α strains in a neutropenic model of candidiasis. Thus, the ability of **a**/α cells to switch phenotypes may be beneficial to *C. tropicalis* as it allows cells to propagate with optimal fitness, but still have the potential to form opaque cells and thereby adapt to different host niches.

Alternatively, the white-opaque switch could have evolved to regulate mating in the ancestor to *C. tropicalis* and *C. albicans*, and *MTL* control of the switch subsequently evolved in *C. albicans* as a means of restricting switching to *MTL* homozygous strains. If the major role of the white-opaque switch is to regulate mating, then fine-tuning of the switching mechanism could have been beneficial to help prevent futile switching to the opaque state in **a**/α cells that are sterile. Certainly, the regulation of mating by the white-opaque switch is stricter in *C. albicans* than in *C. tropicalis*; white cells of *C. tropicalis* undergo appreciable mating frequencies while *C. albicans* white cells do not.

Finally, it is formally possible that *MTL* control of the white-opaque switch was present in the ancestor to *C. albicans* and *C. tropicalis*, but has since been lost in *C. tropicalis.* Experiments will now be required to characterize *C. tropicalis* white and opaque states *in vivo*, and to determine if they exhibit different preferences for host colonization and pathogenesis as has been documented for *C. albicans*.

### A Dual Role for Wor1 in Regulating Phenotypic Switching and Filamentation in *C. tropicalis*

An unexpected outcome of our analysis of white and opaque cells was the discovery of a link between *C. tropicalis* Wor1 and filamentous growth. Overexpression of *WOR1* in *C. tropicalis* **a**, α, or **a**/α cells resulted in increased filamentation relative to wild-type white or opaque cells. *C. tropicalis* Wor1 is therefore a key regulator of filamentation, and this control can be separated from regulation of the white-opaque switch. The role of Wor1 was most clearly manifested in biofilm assays, where increased expression of *WOR1* led to enhanced biofilm formation. This is presumably a direct result of the increase in filamentous growth, as filamentation and biofilm formation are inter-related processes in multiple *Candida* species [35], [36], [50]. Regulation of filamentation by the white-opaque switch has also been noted in *C. albicans*, although here white cells have been shown to be more conducive to undergoing filamentation than opaque cells [23], [24].

The observation that *C. tropicalis* Wor1 induces filamentation shows interesting parallels with the role of Wor1 homologs in more distantly related ascomycete species. In *S. cerevisiae* and *H. capsulatum*, the Wor1 homologs Mit1 and Ryp1, respectively, are both master regulators of filamentation [31], [32]. It has therefore been proposed that the ancestral function of Wor1/Mit1/Ryp1 was to control morphological transitions such as that between yeast and filamentous forms [31]. The discovery that *C. tropicalis* Wor1 promotes filamentation suggests that it has retained the ancestral function of Wor1 in this species.

In contrast to filamentous growth, the white-opaque switch evolved only recently in the ascomycete lineage, probably immediately prior to the divergence of *C. tropicalis* and *C. albicans* [5], [8], [51]. We therefore propose that *C. tropicalis* Wor1 has retained both the ancestral role of the Wor1 family of transcription factors (regulation of filamentous growth), as well as adopting control over the more recently evolved white-opaque switch. In contrast, overexpression of *C. albicans* Wor1 did not promote biofilm formation under the conditions tested (data not shown) and can even inhibit filamentous growth [8]. Further studies on *C. tropicalis* Wor1 will therefore shed light on its role as a key regulator of both morphological switches: the recently evolved white-opaque switch, as well as the ancestral program of filamentous growth.

## Materials and Methods

### Ethics Statement

This study was carried out in strict accordance with the recommendations in the Guide for the Care and Use of Laboratory Animals as defined by the National Institutes of Health (PHS Assurance #A3284-01). Animal protocols were reviewed and approved by the Institutional Animal Care and Use Committee (IACUC) of Brown University. All animals were housed in a centralized and AAALAC-accredited research animal facility that is fully staffed with trained husbandry, technical, and veterinary personnel.

### Media

Yeast extract peptone dextrose medium (YPD), synthetic complete dextrose medium (SCD), and Spider medium were made as described previously [34], [52]. YPD plates containing 200 µg/ml nourseothricin (NAT) were used for selection of strains that were resistant to nourseothricin (Werner Bioagents, Jena, Germany) as previously described [53]. Lee's media containing 12.5 g/L N-acetylglucosamine (Alpha Aesar) was used for switching assays, and Lee's media containing 1.25% glucose was used for biofilm assays. [12], [54]. Lee's media was supplemented with 0.004% histidine when used in experiments with -His strains.

### Plasmids and Strains

*C. tropicalis* strains used in this study are listed in Tables S1 and S2. Gene deletions were constructed by using the *SAT1* flipper strategy as described [55]. Strains were transformed with 1–4 µg of DNA by using a modified electroporation protocol [5]. To delete *WOR1*, *HIS1*, *ARG4*, *MTLa2*, and *MTLα1* genes using the *SAT1*-flipper method, oligonucleotides were used to amplify ∼900 bp of the 5′ and 3′ homologous flanks of each gene. The resulting PCR products were digested with restriction enzymes noted in Table S3 and cloned into the plasmid pSFS2A [55]. The resulting plasmids were digested with restriction enzymes as noted in Table S4 to liberate cassettes containing the 5′ and 3′ gene flanks as well as the *SAT1* selectable marker and used for transformation. Correct genomic integration of transformant colonies was confirmed by PCR of the 5′ and 3′ junctions (for oligonucleotides, see Table S3). The *SAT1* marker was recycled by growing transformants on maltose media at room temperature or 30°C and subsequent replica patching to both YPD and YPD+NAT plates. Alternatively, the *SAT1* marker was recycled by growing cells in liquid YEP + 2% maltose at room temperature for ∼2 days and selected by plating to YPD+ low NAT (10 µg/mL), as previously described [55]. The transformation process was repeated to delete the remaining copy of the gene, and loss of the ORF was confirmed by PCR. To overexpress *WOR1*, fusion PCRs were performed to create a *pTDH3-WOR1* construct, which was cloned into pSFS2A. The plasmid was linearized in the *TDH3* promoter with *Sma*I and transformed. Correct genomic integration was confirmed by PCR. To insert a *SAT1* marker next to the *MTL*, PCR was performed to amplify a sequence immediately upstream of the *MTL*, which was cloned into pSFS2A. The plasmid was linearized within this sequence with *EcoR*I and transformed into *C. tropicalis*. Correct genomic integration next to the *MTL* **a** or *MTL*α locus was confirmed by PCR.

### Mating Assays

Quantitative mating assays between *C. tropicalis* strains were performed as described previously [5], [18]. In brief, *C. tropicalis* cells were taken from Spider plates that had been grown at room temperature for 1–2 days and resuspended in water. Approximately 1×10^7^ cells of each strain were mixed and pipetted onto 0.8-µm pore-size nitrocellulose filters and grown on the surface of Spider medium for 1–3 days at room temperature. Cells were collected from the filters and plated at different dilutions onto His− Arg− media to select for mating products and onto His− and Arg− plates to monitor each parent population. The limiting parent was used to calculate mating frequencies as follows: mating efficiency = conjugants/(limiting parent + conjugants) = the greater of (Arg− His−)/Arg− or (Arg− His−)/His−. Statistical significance was determined using a Student's T-test.

### Phenotypic Switching Assays

White phase cells were inoculated into liquid SCD medium and incubated at room temperature overnight. Cells from this culture were diluted to 0.1 OD_600_ and incubated at 37°C for 5 days. Cultures were diluted in water and plated onto Spider medium or Lee's medium containing N-acetylglucosamine at a concentration of ∼100 colonies per plate. Colonies were examined for opaque sectors after growth at room temperature for 7–10 days.

### RT–PCR

RNA was isolated from cells grown in Spider liquid at 0.8–1.0 OD_600_ using the Ribopure-Yeast Kit (Ambion). RNA was treated with Turbo DNaseI (Ambion), and 2 µg of RNA used for subsequent cDNA generation using the GoScript enzyme (Promega). qRT-PCR was then performed by using the gene specific primers listed in Table S3 with the SYBR Green Kit (Applied Biosystems) and ran on an Applied Biosystems 7300 Real-Time PCR System.

### Microarrays

Cells were harvested after being grown to OD_600_ 1.0–1.2 in Spider medium at room temperature. Cells were collected, flash frozen, and stored at −80°C. For microarrays performed on biofilms, cells were collected after the two-day incubation detailed in the adherence assay. Total RNA was extracted from cell pellets using the RiboPure-Yeast Kit protocol (Ambion). RNA was treated with Turbo DNaseI (Ambion) to eliminate DNA contamination and re-extracted with phenol/chloroform. Aminoallyl-labeled cDNA synthesis and hybridization to custom Agilent *C. tropicalis* microarrays was previously described by Porman *et al*. [5]. Arrays were scanned on a GenePix 4000 scanner (Axon Instruments), data quantified using GENEPIX PRO version 3.0 and normalized using Goulphar (http://transcriptome.ens.fr/goulphar). Data analysis was performed as previously described [5]. GO term analysis was facilitated by CGD (http://candidagenome.org) and Princeton University's Generic GO Term Mapper (http://go.princeton.edu/cgi-bin/GOTermMapper). Array data is available from GEO (accession numbers GSE40179, GSE42517 and GSE43267).

### Microscopy

Digital images of colonies were collected using a Zeiss Stemi 2000-C microscope equipped with an Infinity 2 digital camera and Infinity Analyzer software (Lumenera Corperation, Ottawa, Canada). Differential interference contrast (DIC) of cells were captured using a Zeiss Inverted Microscope (Axio Observer. Z1) fitted with an AxioCam HR. Images were processed with AxioVision Rel. 4.8 (Zeiss, Germany).

For electron micrographs, cells were resuspended in water and attached to poly-L-lysine coated-coverslips. Samples were fixed with 2.5% (w/v) glutaraldehyde in 0.1 M Na-cacodylate buffer, pH 7.4 at 4°C, and washed with 0.1 M Na-cacodylate buffer, pH 7.4. Samples were then treated with 1% aqueous osmium tetroxide in 0.1 M Na-Cacodylate buffer, pH 7.4, at 25°C for 90 minutes, and washed with 0.1 M Na-Cacodylate buffer, pH 7.4. Cells were gradually dehydrated using a gradient ethanol series, dried in a critical point dryer, and coated with 20 nm gold palladium (60∶40) in an Emitech K550 sputter coater. Images were captured with a Hitachi S-2700 scanning electronic microscope with Quartz PCI software.

To quantify filamentation from colonies grown on Spider medium, cells were removed from the center of patches and counted for the fraction of filamentous cells. At least 4 fields of view and 400 cells were counted for each data point. Statistical significance was determined using a Student's T-test.

### Adherence Assays

Cultures were inoculated in 3 ml of Spider medium then incubated at 25°C overnight. 2 ODs of cells were spun down and resuspended in 1 ml of Lee's + Glucose medium and transferred to a well of a 12-well polystyrene plate. Plates were incubated at 25°C for 1–2 days without shaking, then decanted. Each well was washed 3 times with 1 ml of water to remove non-adherent cells. Plates were imaged using a Chemidoc XRS+ with Image Lab software (Bio-Rad). Adherent cells were scraped off the plastic surface and resuspended in 1 ml of water and optical density was determined. For significant differences between data sets, each was tested for normal distribution. A one-way ANOVA was performed on OD_600_ results. Nine representative microscope fields were counted for each condition to determine the fraction of filamentous cells. Statistical significance was determined using a Mann-Whitney pair-wise test due to non-parametric datasets.

### Crystal Violet Staining

Samples were prepared similarly to the adherence assays. Following the 3 washes, 12-well plates were decanted and left to dry for 45 minutes, and subsequently stained with 385 µL of 0.4% aqueous crystal violet per well for 45 minutes. Each well was washed 3 times with 1 mL of water, then destained with 700 µL of 95% ethanol. Finally, 100 µL of each destain solution was transferred to a 96-well plate and diluted 10-fold. Optical density was read at 595 nm using a BioTek Synergy HT plate reader and statistics were performed similarly to the adherence assays.

### XTT Reduction Assay

Samples were prepared similarly to the adherence assays. Following the 3 washes, the plates were decanted, then 315 µL of 1 mg/mL XTT (2,3-bis-(2-methoxy-4-nitro-5-sulfophenyl)-2H-tetrazolium-5-carboxanilide) and 35 µL of 360 µg/mL phenazine methosulfate were added to each well. The plates were incubated at room temperature for 20 minutes, then 100 µL of solution from each well was transferred to a 96-well plate and diluted. Optical density was read at 450 nm using a BioTek Synergy HT plate reader and statistics were performed similarly to the adherence assays.

### Analysis of *C. tropicalis* Fitness *In Vivo*

Female BALB/c mice (18–20 g, Charles River Laboratories) were made neutropenic by intraperitoneal injection of 200 µg anti-Gr-1 mAb (clone: RB6-8C5, BioXCell, West Lebanon, NH) one day prior to infection with *C. tropicalis*. *C. tropicalis* strains were grown overnight in Spider medium at 30°C, diluted to 0.2 OD_600_ in fresh Spider medium and grown at 30°C to log phase. Cells were collected and washed three times in sterile phosphate-buffered saline (PBS). Mice were infected with a mixture of two *C. tropicalis* strains in a 50∶50 ratio with a total inoculum of ∼1.0×10^6^ colony forming units (CFUs) by injection into the tail vein. Each *C. tropicalis* mixture included one isolate auxotrophic for histidine (His) and one isolate auxotrophic for arginine (Arg) biosynthesis. Dilutions of the inoculum were plated onto SCD medium lacking either histidine or arginine to confirm initial cell concentrations. Mice were euthanized 72 hours after infection and kidney and brains isolated. Organs were homogenized through a 70 µm filter and dilutions of the organ suspensions plated onto SCD medium lacking His or Arg. CFUs were counted on each plate to quantify the relative abundance of each strain. Mice where no cells were recovered after infection were excluded from analyses. Statistical significance was determined using a Student's T-test.

## Supporting Information

Figure S1 Scanning Electron Micrographs of *MTL* **a**/a (CAY1504) and *MTL* **a**/α (CAY1513) white and opaque cells and RT-PCR analysis of a1 and α2 expression. (A) White cells were round and exhibited a smooth cell surface, while opaque cells were elongated and typically had a pimpled or uneven surface. Scale bars = 5 µm. (B) RNA was extracted from white (W), opaque (O), *Δ/Δwor1* mutant (m), and *WOR1* overexpressing (OE) cells and examined by RT-PCR for expression of *MTL* **a**1 and *MTL*α2 genes. *ACT1* is a positive control performed with primers against *ACT1* and –RT is RT-PCR performed in the absence of reverse transcriptase with primers against *ACT1*.
(TIF)

Figure S2 Transcriptional profiles of genes regulated by the white-opaque switch in *C. tropicalis* **a**, α, and **a**/α cell types. Panels A–F show genes that passed SAM analysis that were significantly different between white and opaque **a** or **a**/α cells. cDNA prepared from white and opaque states of CAY1511 (**a**/α), CAY1504 (**a**), and CAY1505 (α) in independent experiments was hybridized against a universal reference. Opaque cell gene expression data sets were divided by white cell gene expression data sets. Some genes are regulated by the white-opaque switch in all three cell types (A, D, shared opaque- and white-specific genes). However, many white-opaque regulated genes are unique to *MTL* homozygous cells (**a** cells in panels B and E) or to *MTL* heterozygous cells (**a**/α cells in panels C and F). Numbers of unique and shared genes between **a** and **a**/α white and opaque states are illustrated in panels G and H. Note: a subset of genes that did not pass SAM analysis still appear to be similarly regulated by the switch in each cell type (e.g. middle of panel B and bottom of panel C).
(TIF)

Figure S3 GO Term analysis of genes regulated by the white-opaque switch in C. tropicalis **a** and **a**/α cell types. Graphs represent GO Terms for genes that passed SAM analysis that were significantly different between white and opaque **a** or **a**/α cells. GO Term frequencies for genes regulated by the white-opaque switch in **a** and **a**/α cell types are shown in A and D for shared opaque- and white-specific genes, respectively. GO Term frequencies for white-opaque regulated genes are unique to *MTL* homozygous cells (**a** cells in panels B and E) or to *MTL* heterozygous cells (**a**/α cells in panels C and F) are also presented. * = statistically significant number of genes represented in the GO Term category.
(TIF)

Figure S4 Mating of *C. tropicalis* **a**, α, and **a**/α cell types is regulated by *WOR1* expression. Mating frequency of (A) **a** x α (CAY1503 x CAY1505), (B) **a** x **a**/α (CAY1503 x CAY1511), and (C) α x **a**/α (CAY1505 x CAY1513) white, opaque, or *pTDH3*-*WOR1* cells. Experiments were performed by co-incubating indicated strains on Spider medium for 1 day at room temperature, and then plating cells to selective media to quantify mating frequency. ** p<0.01, * p<0.05. Error bars indicate SD.
(TIF)

Figure S5 Monitoring *MTL* Loss during Mating of *C. tropicalis* **a**/α cell types. (A) Diagram of experimental method for tracking the *MTL* of **a**/α cells during mating. CAY4286 (Arg−) was crossed with CAY1505 (His− sat flip) and plated to selective media (Arg−/His−) after 3 days of incubation on Spider media. Orange S denotes *SAT1* marker from pSFS2A, numbered arrows denote primers used for analysis of products. Primers are listed in Table S3, and marked as FS5-# corresponding to the diagram. (B) Possible outcomes of mating are diagrammed with expected PCR products from primer pairs. (C) Representative PCR analysis of a set of 20 mating products using different combinations of primers is shown. Three mating products contain chromosomes that have undergone recombination (denoted by red arrow), while the majority of the mating products have undergone homozygosis and loss of the *SAT1* marker.
(TIF)

Table S1 Clinical isolates examined in this study.
(XLS)

Table S2 List of *C. tropicalis* strains used in this study. Phenotype not determined unless noted.
(DOCX)

Table S3 Oligonucleotides used in this study. Underlined sequences denote restriction sites.
(DOCX)

Table S4 Plasmids used in this study.
(DOCX)

Table S5 List of **a**/α white-specific genes.
(XLSX)

Table S6 List of **a**/α opaque-specific genes.
(XLSX)

Table S7 List of **a**/α genes down regulated in the white state relative to *Δ/Δwor1* mutants.
(XLSX)

Table S8 List of **a**/α genes up regulated in the white state relative to *Δ/Δwor1* mutants.
(XLSX)

Table S9 List of **a**/α genes down regulated in the opaque state relative to *WOR1* overexpressing strains.
(XLSX)

Table S10 List of **a**/α genes up regulated in the opaque state relative to *WOR1* overexpressing strains.
(XLSX)

Table S11 **a**2 and α1 genes are essential for **a** and α cell mating, respectively. Mating frequency was quantified for wild-type opaque strains and two independent isolates of each mutant. N.D. indicates no mating was detected in these crosses.
(DOCX)

Text S1 Supplemental results.
(DOCX)
