# Supplementary figures and images for: MTL–Independent Phenotypic Switching in Candida tropicalis and a Dual Role for Wor1 in Regulating Switching and Filamentation
Source: PLoS Genet. 2013 Mar 21;9(3):e1003369. doi: 10.1371/journal.pgen.1003369 (PMC3605238; doi:10.1371/journal.pgen.1003369)

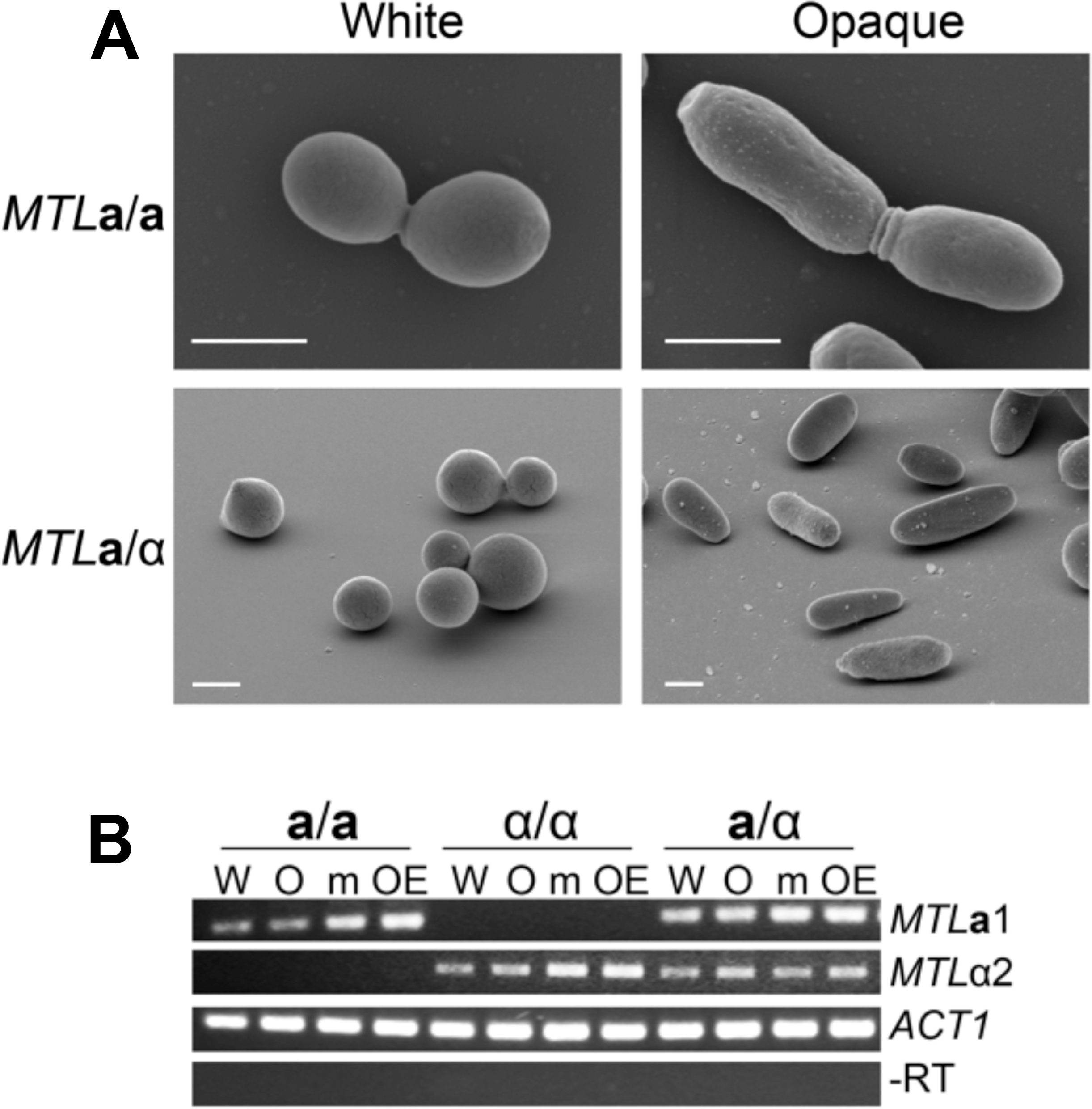

Supplement: Figure S1 — Scanning Electron Micrographs of MTL a/a (CAY1504) and MTL a/α (CAY1513) white and opaque cells and RT-PCR analysis of a1 and α2 expression. (A) White cells were round and exhibited a smooth cell surface, while opaque cells were elongated and typically had a pimpled or uneven surface. Scale bars = 5 µm. (B) RNA was extracted from white (W), opaque (O), Δ/Δwor1 mutant (m), and WOR1 overexpressing (OE) cells and examined by RT-PCR for expression of MTL a1 and MTLα2 genes. ACT1 is a positive control performed with primers against ACT1 and –RT is RT-PCR performed in the absence of reverse transcriptase with primers against ACT1. (TIF) [file pgen.1003369.s001.tif]

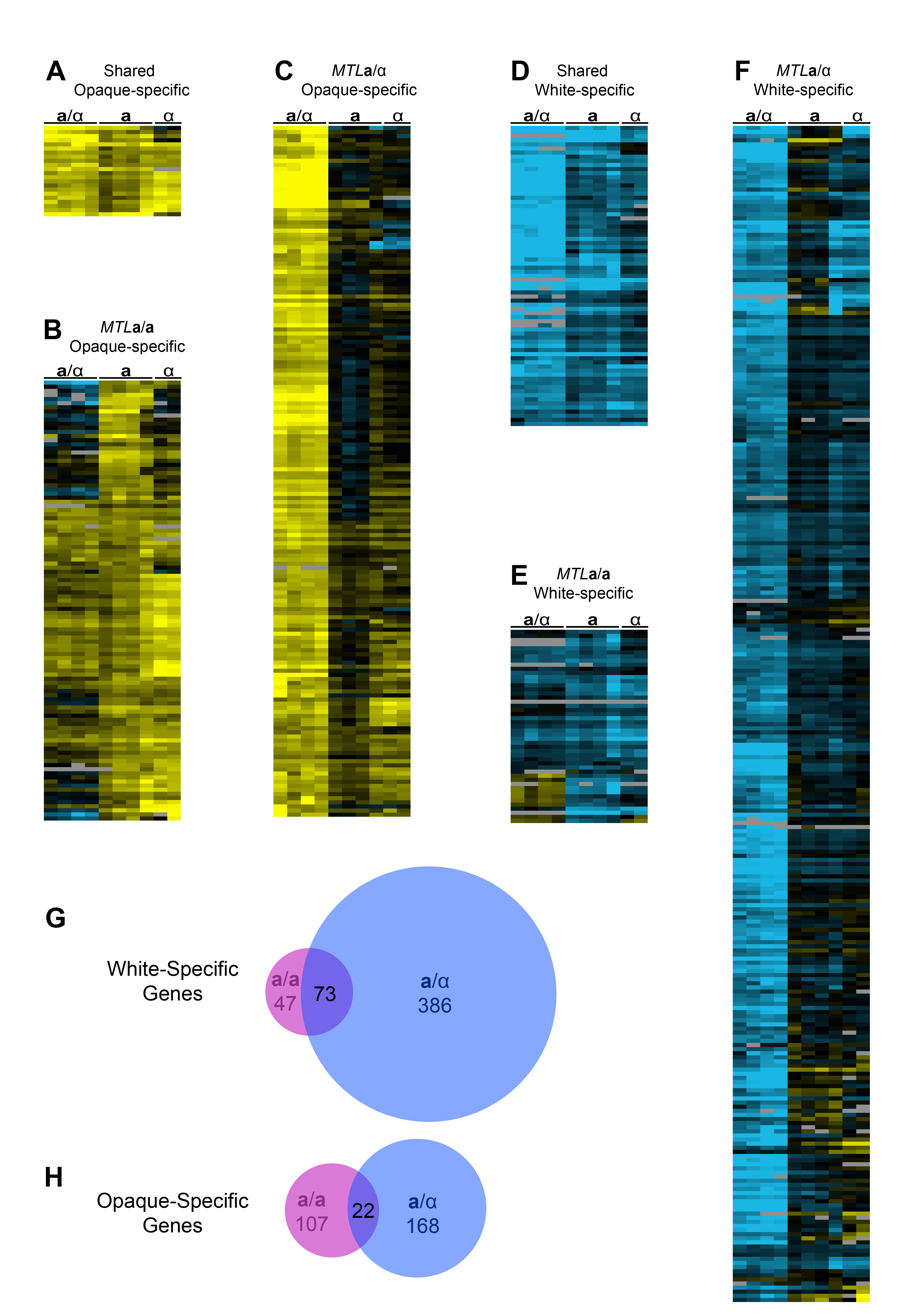

Supplement: Figure S2 — Transcriptional profiles of genes regulated by the white-opaque switch in C. tropicalis a, α, and a/α cell types. Panels A–F show genes that passed SAM analysis that were significantly different between white and opaque a or a/α cells. cDNA prepared from white and opaque states of CAY1511 (a/α), CAY1504 (a), and CAY1505 (α) in independent experiments was hybridized against a universal reference. Opaque cell gene expression data sets were divided by white cell gene expression data sets. Some genes are regulated by the white-opaque switch in all three cell types (A, D, shared opaque- and white-specific genes). However, many white-opaque regulated genes are unique to MTL homozygous cells (a cells in panels B and E) or to MTL heterozygous cells (a/α cells in panels C and F). Numbers of unique and shared genes between a and a/α white and opaque states are illustrated in panels G and H. Note: a subset of genes that did not pass SAM analysis still appear to be similarly regulated by the switch in each cell type (e.g. middle of panel B and bottom of panel C). (TIF) [file pgen.1003369.s002.tif]

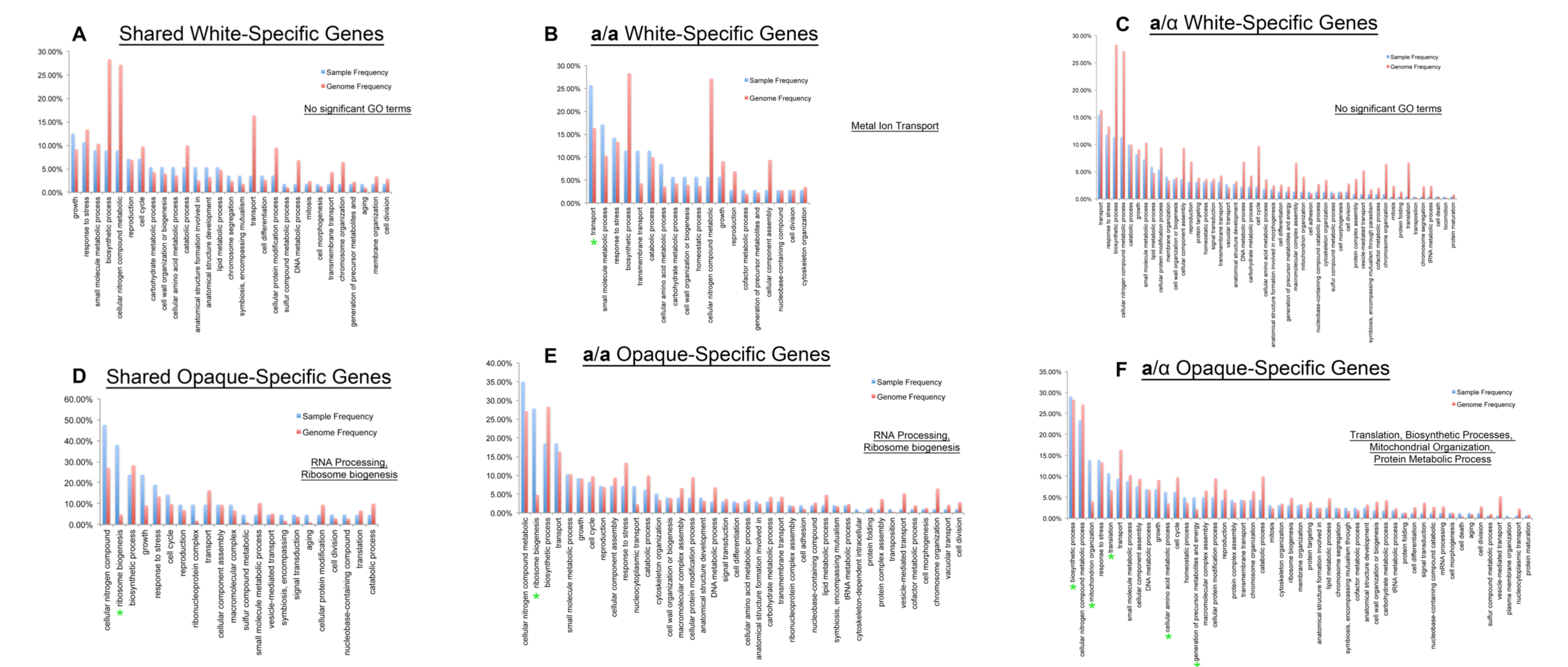

Supplement: Figure S3 — GO Term analysis of genes regulated by the white-opaque switch in C. tropicalis a and a/α cell types. Graphs represent GO Terms for genes that passed SAM analysis that were significantly different between white and opaque a or a/α cells. GO Term frequencies for genes regulated by the white-opaque switch in a and a/α cell types are shown in A and D for shared opaque- and white-specific genes, respectively. GO Term frequencies for white-opaque regulated genes are unique to MTL homozygous cells (a cells in panels B and E) or to MTL heterozygous cells (a/α cells in panels C and F) are also presented. * = statistically significant number of genes represented in the GO Term category. (TIF) [file pgen.1003369.s003.tif]

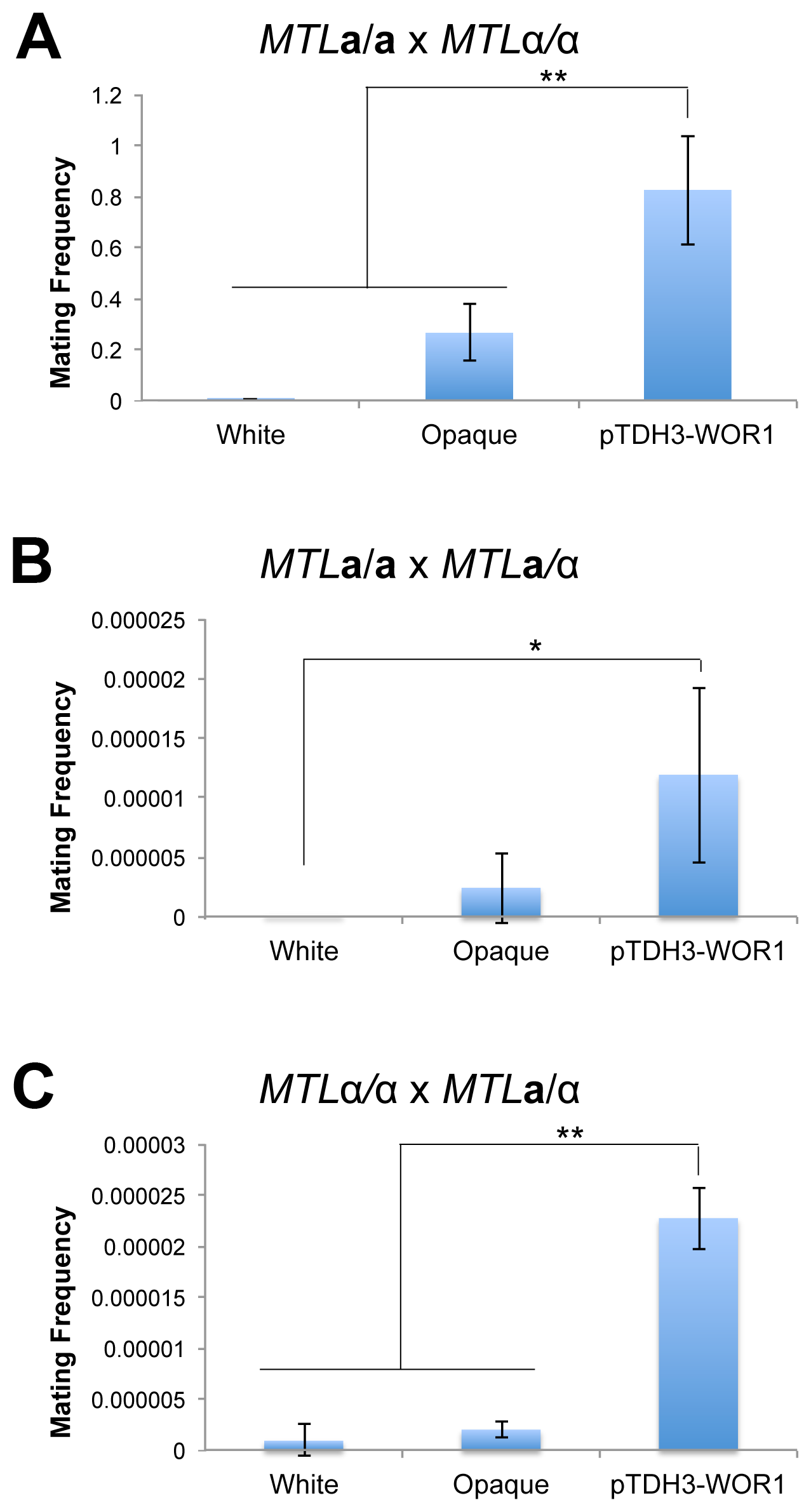

Supplement: Figure S4 — Mating of C. tropicalis a, α, and a/α cell types is regulated by WOR1 expression. Mating frequency of (A) a x α (CAY1503 x CAY1505), (B) a x a/α (CAY1503 x CAY1511), and (C) α x a/α (CAY1505 x CAY1513) white, opaque, or pTDH3-WOR1 cells. Experiments were performed by co-incubating indicated strains on Spider medium for 1 day at room temperature, and then plating cells to selective media to quantify mating frequency. ** p<0.01, * p<0.05. Error bars indicate SD. (TIF) [file pgen.1003369.s004.tif]

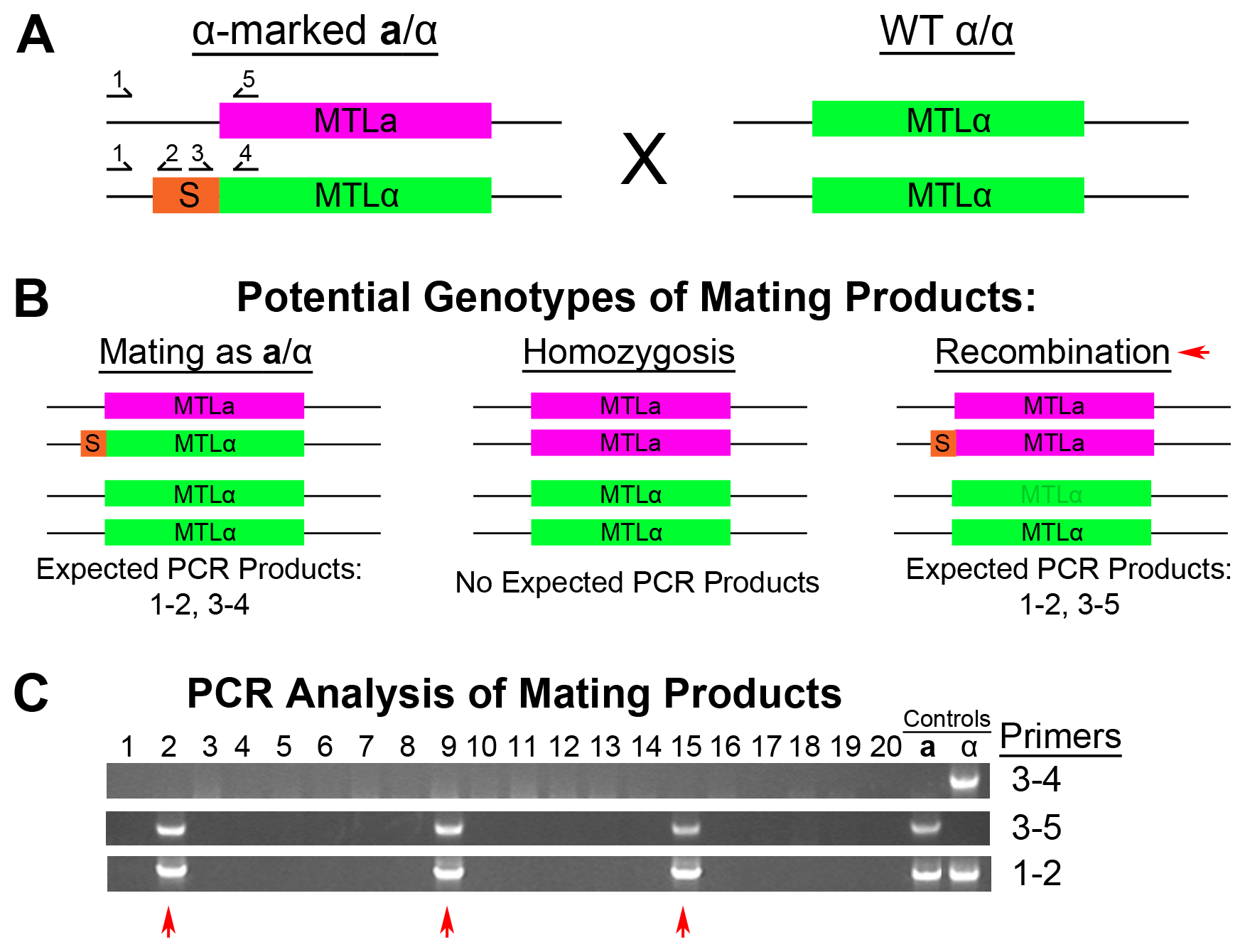

Supplement: Figure S5 — Monitoring MTL Loss during Mating of C. tropicalis a/α cell types. (A) Diagram of experimental method for tracking the MTL of a/α cells during mating. CAY4286 (Arg−) was crossed with CAY1505 (His− sat flip) and plated to selective media (Arg−/His−) after 3 days of incubation on Spider media. Orange S denotes SAT1 marker from pSFS2A, numbered arrows denote primers used for analysis of products. Primers are listed in Table S3, and marked as FS5-# corresponding to the diagram. (B) Possible outcomes of mating are diagrammed with expected PCR products from primer pairs. (C) Representative PCR analysis of a set of 20 mating products using different combinations of primers is shown. Three mating products contain chromosomes that have undergone recombination (denoted by red arrow), while the majority of the mating products have undergone homozygosis and loss of the SAT1 marker. (TIF) [file pgen.1003369.s005.tif]
