# Supplementary material for: MTL–Independent Phenotypic Switching in Candida tropicalis and a Dual Role for Wor1 in Regulating Switching and Filamentation
Source: PLoS Genet. 2013 Mar 21;9(3):e1003369. doi: 10.1371/journal.pgen.1003369 (PMC3605238; doi:10.1371/journal.pgen.1003369)
Supplement: Table S2 — List of C. tropicalis strains used in this study. Phenotype not determined unless noted. (DOCX) [file pgen.1003369.s007.docx]

| **Strain(s)** | **Genotype** | ***MTL*** |
| --- | --- | --- |
| CAY1502 | *his1/his1::SAT1* | **a**/**a** |
| CAY1503 | *arg4/arg4::SAT1* | **a**/**a** |
| CAY3376 | *arg4/arg4::SAT1* (white) | **a**/**a** |
| CAY3378 | *arg4/arg4::SAT1* (opaque) | **a**/**a** |
| CAY1504 | *arg4/arg4::SAT1* (white) | **a**/**a** |
| CAY2275 | *arg4/arg4::SAT1* (opaque) | **a**/**a** |
| CAY1505 | *his1/his1::SAT1* | α/α |
| CAY3391 | *his1/his1::SAT1* (white) | α/α |
| CAY3392 | *his1/his1::SAT1* (opaque) | α/α |
| CAY1509 | *arg4/arg4::SAT1* | α/α |
| CAY1511 | *his1/his1::SAT1* (white) | **a**/α |
| CAY4048 | *his1/his1::SAT1* (opaque) | **a**/α |
| CAY1513 | *arg4/arg4::SAT1* | **a**/α |
| CAY4049 | *arg4/arg4::SAT1* (white) | **a**/α |
| CAY4050 | *arg4/arg4::SAT1* (opaque) | **a**/α |
| CAY2206 | *arg4/arg4 wor1/wor1::SAT1* | **a**/**a** |
| CAY2342 | *his1/his1 wor1/wor1::SAT1* | α/α |
| CAY4043 | *his1/his1 wor1/wor1::SAT1* | **a**/α |
| CAY4012 | *arg4/arg4 wor1/wor1::SAT1* | **a**/α |
| CAY3853 | *arg4/arg4 pTDH3/pTDH3-WOR1::SAT1* | **a**/**a** |
| CAY3976 | *his1/his1 pTDH3/pTDH3-WOR1::SAT1* | α/α |
| CAY4045 | *his1/his1 pTDH3/pTDH3-WOR1::SAT1* | **a**/α |
| CAY3978 | *arg4/arg4 pTDH3/pTDH3-WOR1::SAT1* | **a**/α |
| CAY4289 | *arg4/arg4 a2/a2::SAT1* | **a**/**a** |
| CAY4253/CAY4254 | *his1/his1 a2/a2::SAT1* | **a**/α |
| CAY4251/CAY4252 | *arg4/arg4 a2/a2::SAT1* | **a**/α |
| CAY4290 | *his1/his1 α1/α1::SAT1* | α/α |
| CAY4258/CAY4259 | *his1/his1 α1/α1::SAT1* | **a**/α |
| CAY4256/CAY4257 | *arg4/arg4 α1/α1::SAT1* | **a**/α |
| CAY4286 | *arg4/arg4 MTLα-3’::SAT1* | **a**/α |
