# Supplementary material for: MTL–Independent Phenotypic Switching in Candida tropicalis and a Dual Role for Wor1 in Regulating Switching and Filamentation
Source: PLoS Genet. 2013 Mar 21;9(3):e1003369. doi: 10.1371/journal.pgen.1003369 (PMC3605238; doi:10.1371/journal.pgen.1003369)
Supplement: Table S3 — Oligonucleotides used in this study. Underlined sequences denote restriction sites. (DOCX) [file pgen.1003369.s008.docx]

| Oligo | Name | Sequence |
| --- | --- | --- |
| 1 | *WOR1* (ApaI) -900 | ggagcggggcccCTACTACATTAGTTGTTCCCTG |
| 2 | *WOR1* (XhoI) 0 | gccggcctcgagATAATCTAGACGCCGACATG |
| 3 | *WOR1* (SacII) +2100 | ggagcgccgcggGGTAACTACAGGTGACGAATC |
| 4 | *WOR1* (SacI) +3000 | ggccgcgagctcTTACCCACAAGTGTGTCTAG |
| 5 | *WOR1* 5’ check -950 | GAATCTAAGGCAACAAACGATTTC |
| 6 | *WOR1* 3’ check +3100 | GGTGATTAAAATCACATTCTCGG |
| 7 | *WOR1* 5’ ORF +200 | GACTGATGGAATTTCTTGGTC |
| 8 | *WOR1* 3’ ORF +550 | GCAGCTGTCCATAAACATGG |
| 9 | *HIS1* (ApaI) -900 | ggagcggggcccACCTCAAGAGATGAATGACA |
| 10 | *HIS1* (XhoI) 0 | gccggcctcgagACTCTAAACTATGATTGTATTC |
| 11 | *HIS1* (SacII) +900 | ggagcgccgcggAGTTCATCTGTATAATAGTACA |
| 12 | *HIS1* (SacI) +1800 | ggccgcgagctcTCCACACATTCATAAATGTTCA |
| 13 | *HIS1* 5’ check -950 | CCAAGGACAACACTCGTGCAGT |
| 14 | *HIS1* 3’ check +1900 | CAAATTGATCACTTCTCTAGTCGA |
| 15 | *HIS1* 5’ ORF +200 | AGGGTAATTGTGATTTGGGTA |
| 16 | *HIS1* 3’ORF +600 | CTTGGAAGAAACCAAGTGAG |
| 17 | *ARG4* (ApaI) -900 | ggagcggggcccTGAATGTGTTGGTGGTGATG |
| 18 | *ARG4* (XhoI) 0 | gccggcctcgagGACATTGTTGTTAGAATAAGTT |
| 19 | *ARG4* (NotI) +1400 | ggagcggcggccgcACATTCATTCTCCTTCCCTCTC |
| 20 | *ARG4* (SacII) +2300 | ggccgcccgcggGAATATTGATTGTTGATTGTTG |
| 21 | *ARG4* 5’ check -950 | GACGTTGTTTACCCTTGGTATC |
| 22 | *ARG4* 3’ check +2400 | TTGTGGAATGTAATTTGCACAC |
| 23 | *ARG4* 5’ ORF +200 | TCAGGATTAGAAGAAATTCGTG |
| 24 | *ARG4* 3’ ORF +600 | TGGACTTTGATTAACTCTGGT |
| 25 | *MTLa2* (ApaI) -900 | ggagcggggcccACCATGATTTCGAACATCTGGAA |
| 26 | *MTLa2* (XhoI) 0 | gccggcctcgagATCTTGGTTGCTTTTGATCGTTTT |
| 27 | *MTLa2* (SacII) +700 | ggagcgccgcggGTAGTGCGTACGGTGTGCGAGT |
| 28 | *MTLa2* (SacI) +1600 | ggccgcgagctcATGCTGGCAAGGGAATTTAGTTA |
| 29 | *MTLa2* 5’ check (-950) | GGGATGATTTGCCACAGGAAG |
| 30 | *MTLa2* 3’ check (+1650) | CCATGGGGTAAAAAAAAAATAGAAA |
| 31 | prBT_74 (*MTLa2* 5’) | GCAAGAAACATACTTTCAAAGCAG |
| 32 | prBT_75 (*MTLa2* 3’) | GCCCGTTAAGTCGTTTCTTGGT |
| 33 | *MTLα1* (ApaI) -900 | ggagcggggcccACGTTGTTGGATTATTACAAAAATG |
| 34 | *MTLα1* (XhoI) 0 | gccggcctcgagGGCAATTGGTTTCTTTCAGTGTTA |
| 35 | *MTLα1* (SacII) +700 | ggagcgccgcggTCCTCACTCTTTTCTGACGGTCT |
| 36 | *MTLα1* (SacI) +1550 | ggccgcgagctcGAGTGCTGTCATTGTTGAGAAGTT |
| 37 | *MTLα1* 5’ check (-950) | AAGTCGCTCACAGAACTTTCAATC |
| 38 | *MTLα1* 3’ check (+1600) | GCTAGGATTTGTCAATTATACCCA |
| 39 | prBT_68 (*MTLα1* 5’) | ACTTGAATACGGAATGTTGAATGA |
| 40 | prBT_69 (*MTLα1* 3’) | GGTCTAGGACCGTATATAACCAGTCA |
| 41 | *TDH3* (ApaI) -950 | ggagcggggcccGTTAGTCGTTATGGAGGACA |
| 42 | *TDH3* (0) | TGTTAAAATTTAATTTGTAAGTGATTTGATTT |
| 43 | *WOR1* (0) | aaatcaaatcacttacaaattaaattttaacatccATGTCGGCGTCTAGATTATCATC |
| 44 | *WOR1* (XhoI) +2500 | gccggcctcgagGTCATATACCCAACACCGAAC |
| 45 | prBT_72 (*MTLa1* 5’) | GGATAAAGAGAGCTTAAGTTCAGAAGAG |
| 46 | prBT_73 (*MTLa1* 3’) | AAGTATCATCTGTCTTATCTGATTCCTTC |
| 47 | prBT_70 (*MTL*α2) | TAAAACATTAAGCATAGAGGACAAAGAA |
| 48 | prBT_71 (*MTL*α2) | AACTTCAAATGCAAAATGTAAAACATAC |
| 49 | *ACT1* 5Q | GGTGATGGTGTTACCCACGTT |
| 50 | *ACT1* 3Q | TGTAACCACGTTCAGACAAGATCTTC |
| 51 | *WOR1* 5Q | CCGTCTAATGTTATACCTGCATCAA |
| 52 | *WOR1* 3Q | TTCGTCGTACTTATGGTAATTGTTTTCT |
| 53 | *MTLa1* (0) | gaaagtcaatcaatcactgttttgtactaaaggccATGCTTAAAGCTCAAAAGGAAGAG |
| 54 | *MTLa1* (XhoI) +450 | gccggcctcgagCTAAGTATCATCTGTCTTATCTGA |
| 55 | *MTL*α2 (0) | gaaagtcaatcaatcactgttttgtactaaaggccATGATTAGTCACTGGGATACCCTT |
| 56 | *MTL*α2 (XhoI) +600 | gccggcctcgagTCATTGTGACAATAATTGAGCAAG |
| 57 | *MTL* flank for (ApaI) | ggagcggggcccACACGACACAGTCCAACAGCAGA |
| 58 | *MTL* flank rev (HindIII) | ggccgcaagcttATCACTTCGACCTATTTCTTTTAAAAC |
| 59 | *MTL* flank 5’ check (FS5-1) | AGAACCCGTATGATTTCACGAAAG |
| 60 | pSFS2a *SAT* rev (FS5-2) | CTCAGGGATCACCGAAAT |
| 61 | M13 (-21) for (FS5-3) | TGTAAAACGACGGCCAGT |
| 62 | *MTL*α 3’ check (FS5-4) | GTGACGCCATATTTTCTGGTGC |
| 63 | *MTLa* 3’ check (FS5-5) | GCAAGAAACATACTTTCAAAGCAG |
