# Supplementary material for: MTL–Independent Phenotypic Switching in Candida tropicalis and a Dual Role for Wor1 in Regulating Switching and Filamentation
Source: PLoS Genet. 2013 Mar 21;9(3):e1003369. doi: 10.1371/journal.pgen.1003369 (PMC3605238; doi:10.1371/journal.pgen.1003369)
Supplement: Table S4 — Plasmids used in this study. (DOCX) [file pgen.1003369.s009.docx]

| Plasmid | Name | Oligos used for cloning | Enzyme(s) used for restriction digest of DNA prior to transformation |
| --- | --- | --- | --- |
| pRB29 | *WOR1* deletion plasmid | 1/2, 3/4 | *Apa* I/*Sac* I |
| pRB55 | *HIS1* deletion plasmid | 9/10, 11/12 | *Apa* I/*Sac* I |
| pRB59 | *ARG4* deletion plasmid | 17/18, 19/20 | *Apa* I/*Sac* II |
| pRB288 | *MTL*a2 deletion plasmid | 25/26, 27/28 | *Apa* I/*Sac* I |
| pRB289 | *MTL*α1 deletion plasmid | 33/34, 35/36 | *Apa* I/*Sac* I |
| pRB235 | *pTDH3-WOR1* plasmid | 41/42, 43/44, 41/44 for fusion | *Sma* I |
| pRB290 | *MTL* marker plasmid | 57/58 | *EcoR* I |
