# Supplementary material for: MTL–Independent Phenotypic Switching in Candida tropicalis and a Dual Role for Wor1 in Regulating Switching and Filamentation
Source: PLoS Genet. 2013 Mar 21;9(3):e1003369. doi: 10.1371/journal.pgen.1003369 (PMC3605238; doi:10.1371/journal.pgen.1003369)
Supplement: Table S11 — a2 and α1 genes are essential for a and α cell mating, respectively. Mating frequency was quantified for wild-type opaque strains and two independent isolates of each mutant. N.D. indicates no mating was detected in these crosses. (DOCX) [file pgen.1003369.s016.docx]

|  | **a** × α | **a** × **a**/α | α × **a**/α |
| --- | --- | --- | --- |
| WT Opaque | 0.18 | 2.78 x 10^-6^ | 2.56 x 10^-6^ |
| *Δ****a****2* in **a** | 1.16 x 10^-7^ | N.D. | --- |
| *Δα1* in α | 3.42 x 10^-7^ | --- | N.D. |
| *Δ****a****2* in **a**/α | --- | 6.59 x 10^-6^ | N.D. |
| *Δα1* in **a**/α | --- | N.D. | 4.54 x 10^-6^ |
