# Supplementary material for: MTL–Independent Phenotypic Switching in Candida tropicalis and a Dual Role for Wor1 in Regulating Switching and Filamentation
Source: PLoS Genet. 2013 Mar 21;9(3):e1003369. doi: 10.1371/journal.pgen.1003369 (PMC3605238; doi:10.1371/journal.pgen.1003369)
Supplement: Text S1 — Supplemental results. (DOCX) [file pgen.1003369.s017.docx]

**Supplementary Results**

GO Term analysis of **a**/**a** and **a**/α white- and opaque-specific genes

GO term analysis on white- and opaque-specific genes was used to determine if gene sets indicated any specialized functions for white and opaque **a**/**a** and **a**/α cells.  While both shared and **a**/**a** opaque-specific genes are enriched for genes involved in RNA processing and ribosome biogenesis, the **a**/α opaque-specific gene set also contains genes involved in translation, biosynthetic processes, mitochondrial organization, and protein metabolic processes.  **a**/**a** white-specific genes were enriched for genes involved in metal ion transport, but shared and **a**/α white-specific genes contained no significantly enriched GO terms (see Figure S3).

**a**1/α2 binding sites within the *WOR1* promoter

We analyzed the *WOR1* promoter for **a**1/α2 binding sites by searching for the conserved consensus sequence [1,2] within 5 kb upstream of the *WOR1* gene sequence. We found 3 potential binding sites for **a**1/α2 at positions -2589 (TGTTTTTATTTTTAAGCAG), -2214 (TTCTGTAATTTATAATACT), and -1902 (TGCTAAAAATCCACAT) upstream of the ATG. At present, we cannot confirm that these are bona fide **a**1/α2 sites. However, the presence of putative **a**1/α2 binding sequences suggests that the ability to undergo the white-opaque switch in *C. tropicalis* **a**/α cells is not due to the loss of **a**1/α2 binding sites within the *WOR1* promoter.

Generation of an *MTL* marker to track *MTL* status during **a**/α mating

To track potential *MTL* loss during mating of **a**/α cells, a *SAT1* marker was inserted immediately adjacent to the *MTL*α locus in *C. tropicalis*. As shown in Figure S5A, an **a**/α strain containing the *SAT1* marker upstream of the *MTL*α locus (Arg- strain) was mated with an unmarked α/α strain (His- strain). Mating products were selected on Arg- His- medium and tested by PCR for the presence of the marker (Figure S5B and C). Out of 52 mating products tested from 4 independent experiments, none still contained the marker next to the *MTL*α locus (a subset of products are shown in Figure S5C). Interestingly, 4 out of the 52 products (7.7%) had undergone recombination so that the *SAT1* marker was now adjacent to the *MTL***a** locus. This result indicates that recombination events immediately adjacent to the *MTL* can take place at an appreciable frequency in *C. tropicalis*. However, the majority of mating products (48/52) had lost the *SAT1* marker, presumably either by loss of the chromosome containing the *MTL*α locus or by recombination outside of the *SAT1* marker region.

Regulation of mating in *C. tropicalis* by **a**2 and α1 transcription factors

In *C. albicans*, mating of **a** cells is dependent on the **a**2 transcription factor, while mating of α cells is dependent on the α1 transcription factor [3]. The roles of **a**2 and α1 in *C. tropicalis* mating were therefore examined, and **a**2 was found to be essential for **a**-cell mating, while α1 was essential for α-cell mating (Table S11), similar to their roles in *C. albicans* [3]. Mating of *C. tropicalis* **a**/α cells was also dependent on **a**1 and α2 transcription factors, as **a**/α cell mating with α cells required **a**2, while **a**/α cell mating with **a** cells required α1 (Table S11). It is therefore apparent that *C. tropicalis* **a**/α cells behave like **a** cells when mating with α cells, and like α cells when mating with **a** cells. We presume that loss or recombination at the *MTL* locus occurs prior to **a**/α cell mating, subsequently allowing conventional mating with **a** or α partners to occur.

Comparison of white- and opaque-specific genes in *C. tropicalis* and *C. albicans* Genes regulated by the white-opaque switch in **a**/α *C. tropicalis* cells were compared to those regulated by the switch in *C. albicans* **a**/**a** cells [3]. This approach is similar to a previous analysis between *C. tropicalis* and *C. albicans* white and opaque **a**/**a** cells [4]. Interestingly, of the 180 *C. tropicalis* **a**/α opaque-specific genes with homologs in *C. albicans*, only 5 (2.8%) were also opaque-specific in *C. albicans*, while 4 (2.2%) were white-specific in *C. albicans* (Table S6). Similarly, of the 281 *C. tropicalis* **a**/α white-specific genes with homologs, 23 (8.2%) were also white-specific while 20 (7.1%) were opaque-specific in *C. albicans* (Table S5). These results indicate that most of the genes regulated by the white-opaque switch in *C. tropicalis* **a**/α cell types are not shared with those that are switch-regulated in *C. albicans*. Furthermore, *C. tropicalis* genes regulated by the white-opaque switch in **a**/α cells have significantly less overlap with switch-regulated genes in *C. albicans* (6% for both white and opaque genes) than do those in *C. tropicalis* **a**/**a** cells (25% for white- and opaque-specific genes). Thus, while the white-opaque switch in *C. tropicalis* **a**/α cells regulates an increased number of genes relative to **a**/**a** cells, this gene set is more divergent to those regulated by the switch in *C. albicans*. Taken together, these results demonstrate that the white-opaque switch regulates distinct sets of genes between different cell types and between different *Candida* species.

Comparative analysis of *Δ/Δwor1* mutant cells with white cells, and *pTDH3-WOR1* expressing cells with opaque cells

To compare *Δ/Δwor1* mutant cells and white cells, and *pTDH3-WOR1* expressing cells and opaque cells, SAM analyses were performed on the corresponding microarray data (Tables S7-S10). Interestingly, we found that genes upregulated in *pTDH3-WOR1* strains relative to opaque strains were enriched for “genes involved in biofilm formation by a single species on an inanimate substrate” based on their function in *C. albicans*. These included *BCR1*, a master regulator of biofilm formation, *CRZ2*, a transcription factor that is involved in biofilms, and *CSR1*, a zinc-finger protein involved in regulation of biofilm matrix (see Table S9). It is therefore possible that some of these genes contribute to the increased biofilm formation observed in *C. tropicalis WOR1* overexpression strains (see Figure 6). There were also several genes that were down regulated in the *C. tropicalis wor1* mutant strain versus the white strain, including *STB5*, which has sequence-specific DNA binding transcription factor activity and could potentially play a role in regulation of the switch by Wor1 (see Table S7).

**References**

1. Booth LN, Tuch BB, Johnson AD (2010) Intercalation of a new tier of transcription regulation into an ancient circuit. Nature 468: 959-963.

2. Butler G, Rasmussen MD, Lin MF, Santos MA, Sakthikumar S, et al. (2009) Evolution of pathogenicity and sexual reproduction in eight *Candida* genomes. Nature 459: 657-662.

3. Tsong AE, Miller MG, Raisner RM, Johnson AD (2003) Evolution of a combinatorial transcriptional circuit: a case study in yeasts. Cell 115: 389-399.

4. Porman AM, Alby K, Hirakawa MP, Bennett RJ (2011) Discovery of a phenotypic switch regulating sexual mating in the opportunistic fungal pathogen *Candida tropicalis*. Proc Natl Acad Sci U S A 108: 21158-21163.
